# Rapid and up-scalable manufacturing of gigahertz nanogap diodes

**DOI:** 10.1038/s41467-022-30876-6

**Published:** 2022-06-07

**Authors:** Kalaivanan Loganathan, Hendrik Faber, Emre Yengel, Akmaral Seitkhan, Azamat Bakytbekov, Emre Yarali, Begimai Adilbekova, Afnan AlBatati, Yuanbao Lin, Zainab Felemban, Shuai Yang, Weiwei Li, Dimitra G. Georgiadou, Atif Shamim, Elefterios Lidorikis, Thomas D. Anthopoulos

**Affiliations:** 1 Physical Science and Engineering Division, KAUST Solar Center (KSC), King Abdullah University of Science and Technology (KAUST), Thuwal, 23955-6900 Saudi Arabia; 2 Computer, Electrical and Mathematical Science and Engineering Division, King Abdullah University of Science and Technology (KAUST), Thuwal, 23955-6900 Saudi Arabia; 3 Electronics and Computer Science, University of Southampton, Southampton, SO171BJ UK; 4 Department of Materials Science and Engineering, University of Ioannina, Ioannina, 45110 Greece; 5 University Research Center of Ioannina (URCI), Institute of Materials Science and Computing, 45110 Ioannina, Greece

**Keywords:** Nanoscience and technology, Materials science

## Abstract

The massive deployment of fifth generation and internet of things technologies requires precise and high-throughput fabrication techniques for the mass production of radio frequency electronics. We use printable indium-gallium-zinc-oxide semiconductor in spontaneously formed self-aligned <10 nm nanogaps and flash-lamp annealing to demonstrate rapid manufacturing of nanogap Schottky diodes over arbitrary size substrates operating in 5 G frequencies. These diodes combine low junction capacitance with low turn-on voltage while exhibiting cut-off frequencies (intrinsic) of >100 GHz. Rectifier circuits constructed with these co-planar diodes can operate at ~47 GHz (extrinsic), making them the fastest large-area electronic devices demonstrated to date.

## Abstract (web summary)

The incoming internet of things technology requires mass production of radiofrequency electronics. Here, Anthopoulos et al. report a self-forming nanogap method for manufacturing Schottky diodes, operating at 47 GHz, over arbitrary size substrates.

## Introduction

Fifth generation (5 G) mobile networks are now a commercial reality and research towards sixth generation (6 G) technologies, operating at frequencies above 95 GHz, is well underway^1^. This will extend the use of augmented and virtual reality in combination with the emerging internet of things (IoT) platform^1^. Both 5 G and 6 G demand high frequency devices, such as Schottky diodes, transistors, antennas, and switches, all of which at distinctively low cost to enable their projected massive deployment^1–3^. Schottky diodes are ubiquitous critical elements in radio frequency (RF) electronics, such as rectifier circuits, frequency multipliers, and mixers^2,4^. Current state-of-the-art Schottky diode technologies are based on Si and III–V semiconductors relying on established and highly sophisticated fabrication methods^2^. Unfortunately, these come with major technological limitations including, incompatibility with flexible substrates and large-area production, limited-throughput and high-temperature processing. As a result, the massive adoption of existing RF diode technologies in large-area electronics remains challenging.

RF Schottky diodes made of metal oxide semiconductors have been attracting increasing attention in recent years due to their high charge carrier mobility, eco-friendly and inexpensive materials, ease of processing, mechanical compliance and compatibility with large-area polymer substrates^5–8^. The key parameters that ultimately dictate the operating frequency of a Schottky diode are the junction capacitance (C_j_) and the device series resistance (R_s_)^2^. To achieve GHz operation in Schottky diodes, both ultra-small capacitance (<pF) and low series resistance are thus necessary. In conventional vertical sandwich devices, the overlapping capacitance between the top and bottom electrodes is a limiting factor for high frequency operation, which can be, albeit partially, addressed via downscaling of the diode’s size. The pinholes that often exists in thin layers of the active semiconductor impose additional limitation on the reliability and manufacturing yield of such devices. Recently, Aimin Song et al.^5^, demonstrated 6.3 GHz operation in IGZO Schottky diodes featuring an RF mesa structure composed of electrodes and semiconductor layers of optimized area and thickness. In a separate study, Zhang et al.^4^, developed micron-size co-planar diodes utilizing the two-dimensional (2D) semiconductor MoS_2_ that was spatially tailored to exhibit semiconducting and metallic properties that form the channel. The ensuing planar diodes exhibited C_j_ values below 10 fF and operated at frequencies above 10 GHz. Although very promising, fabrication of such devices remains challenging, as it relies on elaborate engineering of the MoS_2_ involving sophisticated patterning steps via e-beam lithography.

Adhesion lithography (a-Lith) has recently been used to alleviate some of the limitations encountered by conventional vertical Schottky diodes^7,9–11^, by enabling the development of coplanar junction architectures with ultra-low capacitance and short carrier transit times^10,11^. A wide range of other planar devices including non-volatile memories^12^, photodetectors^13^, self-aligned-gate thin-film transistors (SAG-TFTs) and light-emitting diodes (LEDs)^14^, all relying on planar nanogap electrodes, have also been demonstrated using a-Lith. In conventional a-Lith, octadecyl phosphonic acid (ODPA) is used as self-assembled monolayer (SAM) to modify the surface energy of the first electrode (M1) and reduce the adhesion of the subsequently processed second metal electrode (M2). The latter is then peeled off (from the M1-SAM/M2 interface) with adhesive tape or glue, leaving behind adjacent M1 and M2 electrodes separated by a nanogap. This manual peel-off step, however, impacts the nanogap size and uniformity, leading to measurable variations between devices^9,11^, thus impairing the adoption of this technology in a fully automated industry-relevant fabrication processes.

We show that the peel-off step can be avoided altogether by adopting a Ti-Pt bimetal combination as M2. The bimetal M2 spontaneously delaminates during deposition at its interface with M1-SAM while remaining strongly attached to the substrate. This leads to consistent formation of sub-10-nm nanogaps and enables reliable large-area manufacturing of coplanar metallic nanogaps. We furthermore deploy flash lamp annealing (FLA) for the rapid conversion of the solution processed metal oxide semiconductor (Indium Gallium Zinc Oxide, IGZO) across the nanogap channels. Unlike conventional thermal annealing which relies on high temperature (≥400 °C) over prolonged times (>45 min)^15^, FLA enables treatment of metal oxide films on large areas^16^ at reduced thermal budget over temperature-sensitive substrate materials^17^.

## Results and discussion

**Fig. 1:**
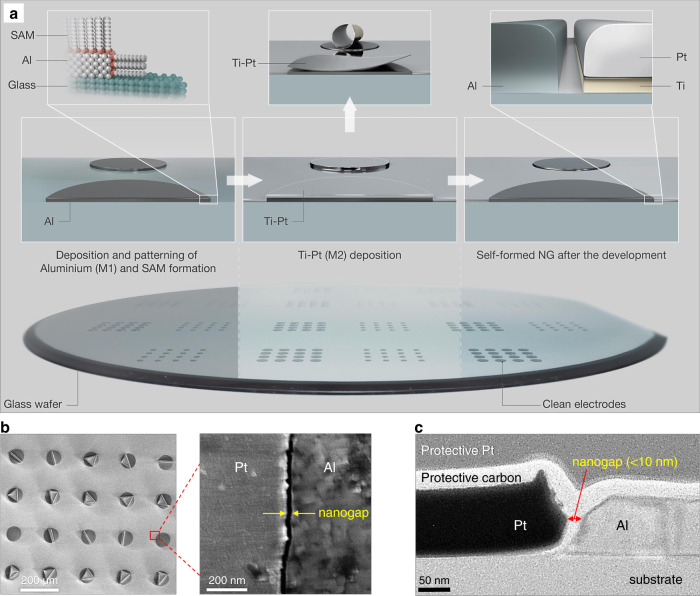
Fabrication of self-forming nanogap electrodes. **a** Schematic illustrations of wafer scale nanogap electrodes fabrication. The key steps involved the patterning and selective SAM formation on Al electrodes (left) followed by Ti-Pt deposition (middle), resulting the self-peeling of Ti-Pt films on Al/SAM surfaces (right). **b** SEM image displaying the self-peeling of Ti-Pt only on top of Al/SAM surfaces and reveals nanogap between Al and Ti-Pt metals. **c** High-resolution cross-sectional TEM images showing the nanogap (<10 nm) between Al/Ti-Pt electrodes.

The process steps for fabricating co-planar arrays of aluminum (Al)/titanium - platinum (Ti-Pt) nanogap electrodes are shown in Fig. 1a and Supplementary Fig. 1. The poor adhesion of Ti-Pt (M2) on M1/SAM due to the hydrophobic alkyl tail and methyl end group (Supplementary Fig. 2) and the internal stresses within M2 enables the explicit self-peeling of the second layer (M2), as shown in Supplementary Fig. 3. This self-peeling either leads to instantaneous full removal of the Ti-Pt films or causes them to roll up into tubes and various shapes, as shown in Supplementary Fig. 4. The complete removal of any remaining M2 is achieved by a nitrogen stream or by immersing the entire substrates into a liquid (acetone, iso-propyl alcohol or DI water) with gentle agitation (movie S1). M2 residues can then be collected and recycled. Arrays of circular, interdigitated, and elongated bar structures were fabricated on 4-inch glass wafers (Supplementary Fig. 5). The detailed and specific dimensions of the diode structures used in this study are illustrated in Supplementary Fig. 6. The inter-electrode distance, i.e., nanogap length, *L*, is less than 18.7 nm, as elucidated from top-view scanning electron microscopy (SEM) images (Fig. 1b and Supplementary Fig. 7, 8). However, as determined by cross-sectional transmission electron microscopy (TEM) (Fig. 1c), *L* can be smaller than 10 nm.

**Fig. 2:**
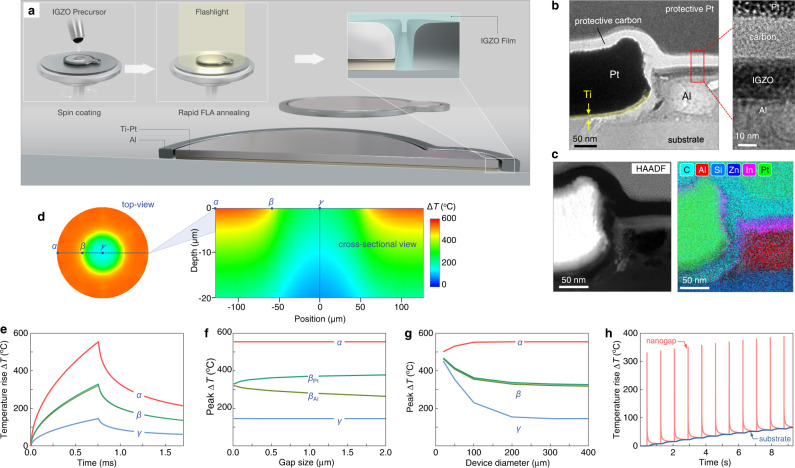
Fabrication of co-planar Al/IGZO/Ti-Pt diodes via flash lamp annealing (FLA) route. **a** Schematic illustration of solution processing of IGZO films on nanogap electrodes. **b**, **c** HR-TEM cross-sectional image and the corresponding EELS mapping that shows the In, Zn, and O elements within the nanogap space. **d** Temperature color map profile on Ø300 µm device displays the temperatures on Ti-Pt (α), Al (γ) and at the edges of the electrodes (β). **e** Time dependent temperature rise (∆T) plot in α, β, γ points. **f** The peak temperature rise as a function of lateral gap separation illustrate that at lower gap size (<30 nm) the temperature difference in channel is negligible. **g** Shows the peak temperature rise (∆T) in α, β, and γ regions with respect to inner metal diameter and it converges after 200 µm. **h** Depicts the full transient including the first 11 pulses arriving at 1.2 Hz repetition rate and 750 µs pulse duration.

To investigate the suitability of the self-forming asymmetric nanogap electrode for the fabrication of Schottky diodes, we employed IGZO as the n-type semiconductor. In the resulting co-planar Al/IGZO/Ti-Pt device structure, the Al (M1) electrode serves as the Ohmic contact due to its low work function (~4.2 eV) and the good matching with IGZO’s conduction band minimum, while the bimetallic Ti-Pt (M2) forms a Schottky contact due to the large work function of Pt (≈5.8 eV)^8^. IGZO deposition was implemented in two steps: (i) the precursor deposition was performed via a sol-gel route, and (ii) the sample was subjected to FLA in ambient atmosphere. These key processing steps are schematically illustrated in Fig. 2a. As seen from Fig. 2b, c and Supplementary Fig. 9–11, the formed IGZO layer appears to fill the nanogap although variations in the concentration of the various relevant elements across it, can be observed. This maybe be the result of IGZO partially filling the nanogap and/or to extrinsic effects induced during lamella preparation (i.e. ion milling and polishing) where sample damage may occur. Despite these variations, we conclude that the IGZO layer fills the nanogap and forms contacts with the M1 and M2 electrodes. Opto-thermal simulations reveal the effect of FLA process parameters on the temperature transients developed. Figure 2d displays the temperature profile on Ti-Pt (α) and Al electrodes (γ) and at the metal edges (β) in the nanogap. As shown in Fig. 2e, the temperature on M2 (Ti-Pt) is higher than on M1 (Al) due to its larger percentage of light absorption. Despite this difference, however, the proximity of the electrodes (<10 nm) enables an almost uniform temperature distribution within nanogap (β) (Supplementary Fig. 12 and Table 1). Significant gradients in peak temperature-rise within the gap are only found for gaps exceeding 100 nm (Fig. 2f). The latter finding highlights the unique advantage of the short (<16 nm) inter-electrode nanogap. Temperatures in and out of the nanogap depend on the device area (size), converging for device diameters exceeding 200 µm (Fig. 2g). The effect of repeated FLA pulses at $v=1.2{{{{{\rm{Hz}}}}}}$ reaches saturation after 10 pulses, yielding instantaneous $\varDelta {T}_{{peak}}\cong 360^\circ {{{{{\rm{C}}}}}}$ and back-of-the substrate $\varDelta {T}_{{back}}\cong 35^\circ {{{{{\rm{C}}}}}}$ (Fig. 2h). The role of FLA parameters on the diode performance are shown in Supplementary Fig. 13. Overall, FLA facilitates targeted, rapid, and precise energy delivery in the nano-channel while leaving the substrate intact.

**Fig. 3:**
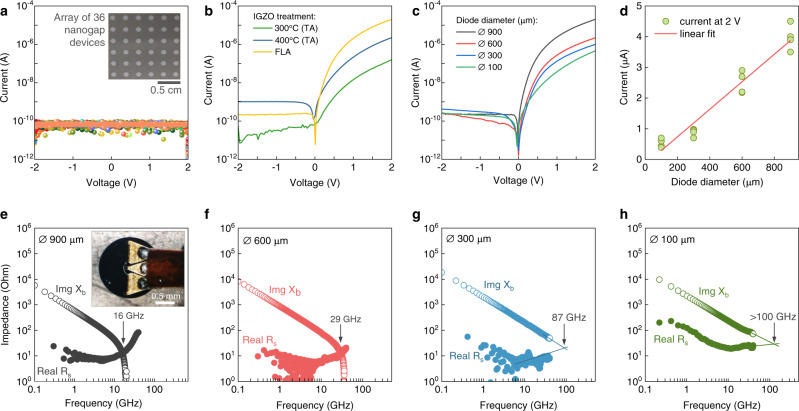
Electrical and S-parameters (S_11_) measurements. **a** I–V characteristics of one complete array of 36 devices consisting of empty Al/Ti-Pt nanogap electrodes (inset photograph). The consistent current level below 0.1 nA implies complete electrical insulation between both metals. **b** I–V characteristics of thermally annealed and Flash Lamp Annealed (FLA) Al/IGZO/Ti-Pt diodes. The FLA processed diodes show better rectification, less hysteresis, and a turn-on closer to 0 V compared to the thermally annealed diodes. **c** I–V characteristics of FLA diodes where the current is scaling up with the diode’s diameter. **d** The current at 2 V for several diodes, which is scaling with diode’s diameter. The linear fit to the experimental data (R^2^ = 0.997) validates the use of diode’s area as the product of circumference (πd) times the thickness of Ti-Pt (M2) electrode (100 nm). **e**–**h** Frequency dependent impedance of diodes extracted from S_11_ measurements. The inset in (**e**) shows a ground-signal-ground (GSG) probe in contact with a co-planar diode.

I–V characteristics of empty nanogaps (Fig. 3a) show excellent electrical isolation (current levels of <10^−10 ^A) for all devices contained in an array of 36 individual 900 µm diameter diodes (inset photograph), suggesting 100% yield in nanogap formation. After IGZO coating and FLA process, the diodes showed n-type behavior, high rectification, and ultra-low reverse current (~10^−10 ^A). In comparison, reference devices that were prepared via thermally annealing at two different temperatures (300 and 400 °C) required long annealing times (~45 min) and showed hysteresis, lower rectification and higher turn-on voltage (Fig. 3b). The FLA diodes exhibit rectification ratio of >10^4^ (Fig. 3c) while the forward current (at 2 V) scales linearly with the diode’s diameter (Fig. 3d). The FLA diode junction parameters, such as series resistance (*R*_S_), barrier height (*Φ*_B_), ideality factor (*n*), effective Richardson constant (A^*^), and built-in potential (*V*_bi_) were extracted from *I-V*, *I-V-T*, and *C-V* measurements (Supplementary Figs. 14–17) with results summarized in Supplementary Table 2.

The impact of air on the operating characteristics of as-prepared Al/IGZO/Ti-Pt diodes was also investigated since IGZO is known to be prone to oxygen and water molecules, which can cause degradation in the device performance (see Supplementary Fig. 15)^18^. Indeed, the Schottky diodes show increased reverse current with clockwise hysteresis when measured in ambient air at 25 °C with a relatively humidity of ≈55%. Applying a layer of the epoxy-based negative photoresist SU-8 atop passivates the diodes and leads to consistent I–V characteristics even after prolonged exposed to ambient atmosphere for 3 months (see Supplementary Fig. 15c). These results highlight the compatibility of simple to implement passivation layers for stabilizing the operation of our Schottky diodes. The bias stability of the planar IGZO devices was also investigated. Non-encapsulated diodes show reverse breakdown voltage of ≈5 V beyond which the current increases sharply. (Supplementary Fig. 18a). Similar findings were reported for similar planar nanogap diodes based on different semiconductors^9,10^. Despite the relatively low breakdown voltage, however, we believe our planar IGZO diodes are well suitable for a range of low-power RF applications such as RF mixers, detectors, logic circuits and wireless energy harvesters.

**Fig. 4:**
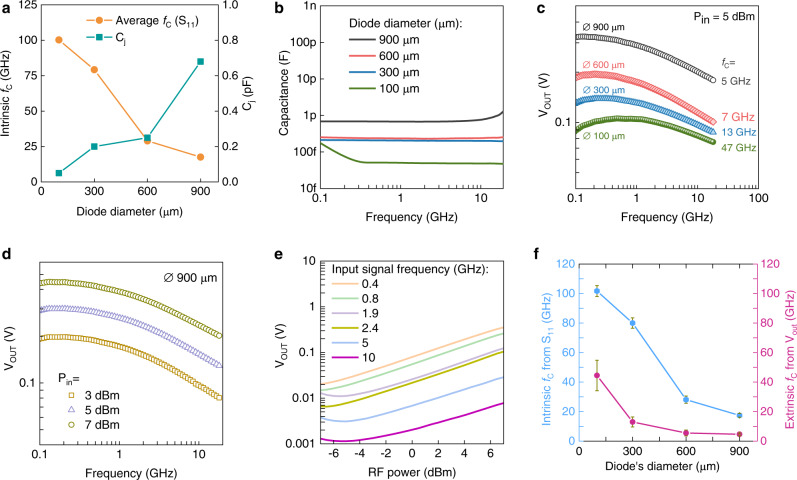
High-frequency capacitance and rectifier measurements. **a** The average intrinsic cut-off frequency and junction capacitance (C_j_) of several diodes (for each diameter) measured from S_11_ one-port measurements. The cut-off frequency is increasing as the diode’s diameter is reduced. On the other hand, C_j_ (measured at 10 GHz) is reducing for decreasing diameters. **b** The junction capacitance of Al/IGZO/Ti-Pt diodes extracted from S_11_ measurements in the range from 0.1 GHz to 18 GHz. In all cases, C_j_ displays ultra-small values (<1 pF). **c** The rectified voltage output vs. input frequency at 5 dBm power input. The extrinsic cut-off frequencies were extracted at the half power point. The power-dependent (**d**) and frequency-dependent (**e**) voltage output of a diode with a diameter of 900 µm. **f** Dependence of the intrinsic cut-off frequency, (f_C,int_, from S_11_), and extrinsic cut-off frequency, (*f*_C,ext_, from voltage output measurements), measured for 10 diodes per diameter indicating the clear increase in f_C_ with reducing diode width. The error bar indicates the standard deviation of the cut-off frequency of the diodes.

For RF wireless energy harvesting and RF identification tags, the antenna-diode rectifier circuits ultimately dictate the frequency of operation, power conversion efficiency, and cost^2^. For instance, the high nonlinearity (>3) and current responsivity (6–8 AW^−1^) of our diodes (Supplementary Fig. 18b, c) are critical factors for RF applications^4^. The frequency response was measured with a one-port scattering measurement setup (Supplementary Fig. 19) using high-frequency input signals and extracting frequency dependent reflection coefficient (S_11_) and diode impedance. The intrinsic cut-off frequency, *f*_*C,int*_, can be estimated from the intersection of the real (*R*_S_, series resistance) and imaginary (*X*_C_, reactance) part of the impedance (Fig. 3e–h). Notably, the series resistance extracted from the real part of the impedance represents the effective series resistance (R_se_) of the device (mainly contact resistance) and excludes the resistance associated with the junction’s depletion region. As such, its value is orders of magnitude lower than that extracted from the DC current-voltage characteristics of the diode^5^. Surprisingly, the R_se_ for diodes with large diameters (600 and 900 µm) remains similar instead of decreasing with increasing nanogap width. The RF current distribution profile simulations presented in Supplementary Fig. 20 provide an explanation to this anomaly. As the diameter of the diode increase from 100 to 900 μm, the current distribution profile appears confined near the feeding point (i.e. the location in the middle electrode where the RF signal is launched) and does not spread uniformly across the whole electrode. As a result, for larger size diodes the measured R_se_ ceases to scale with the width and follows a more convoluted relationship. The rapid change in the impedance seen beyond the cut-off frequency point is most likely the result of resonances in our circuit. Similar behavior was reported recently for nanogap diodes based on different metal oxides and organic semiconductors^9,10^. The intrinsic cut-off frequency values extracted from Fig. 3e–h range between 16 GHz, for the larger diodes (900 μm), to over 100 GHz, for the smallest diameter diode (100 μm). The latter observation is attributed mainly to the reduction in diode junction capacitance (*C*_j_) and the series resistance (*R*_S_) (Supplementary Table 3). Several diodes per channel diameter were measured (Supplementary Fig. 21, 22), from which the average *f*_*C,int*_ and *C*_j_ were calculated and summarized in Fig. 4a, b, respectively.

**Fig. 5:**
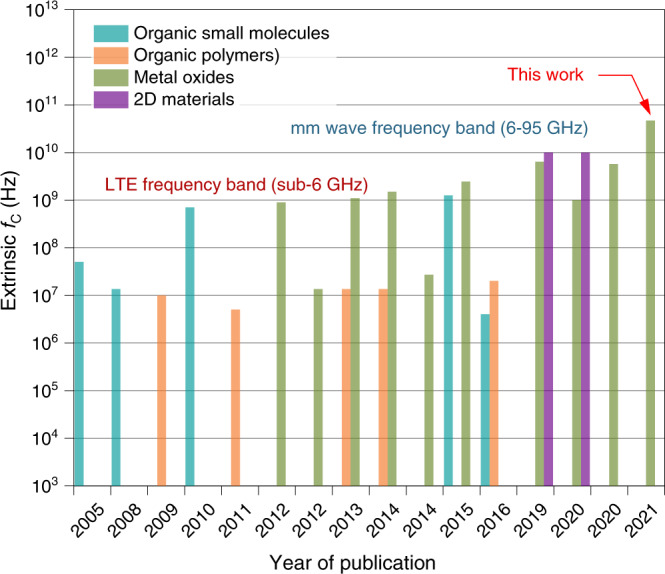
Comparison of state-of-the-art Schottky diodes based on different semiconductor technologies and processing methods. The plot compares the extrinsic cut-off frequency, (f_C_) of our current diodes (This work) with previously reported Schottky diodes fabricated using different semiconductor materials (i.e. organic small molecules, organic polymers, metal oxides and 2D materials), via vacuum and solution processing methods. The literature data used for this figure are summarized in Supplementary Table 4.

In real-life applications, the extrinsic cut-off frequency, *f*_*C,ext*_, of the diode is more relevant as it is influenced by all device components. It can be estimated from the −3 dB point, i.e. the frequency at which the power drops by 1/2 (frequency at which voltage output, V_OUT_, drops to 1/$\sqrt{2}$) of its peak value^2^. The rectified DC output voltage as a function of frequency was measured using a half-wave rectifier setup, comprising a bias tee, Al/IGZO/Ti-Pt diode and a 10 MΩ load resistor, R_L_ (Supplementary Fig. 23). The −3 dB point projection and the resulting extrinsic cut off frequencies for all diodes are shown in Fig. 4c. As expected, the output voltage (V_OUT_) scales up both with an increase of the diode’s active area (Fig. 4c) and input power (Fig. 4d). The output voltage as a function of input RF power follows a square law at low input power and square root law at higher input power. The corresponding two linear regions are clearly discernible up to 5 GHz in Fig. 4e. The evolution of the intrinsic (from S_11_ measurements) and extrinsic (from rectifier circuit measurements) cut-off frequencies with increasing nanogap diameter (extracted from 10 diodes per diameter), are shown in Fig. 4f. We observed small standard deviation in the cut-off frequencies for diodes with diameter in the range 300–900 µm. The slightly higher standard deviation observed in the *f*_C_ of the 100 µm diameter diodes, could be the result of slightly different preparation and testing conditions. The large difference observed between the intrinsic and the extrinsic *f*_C_ values are most likely attributed to parasitic losses associated with the rectifier circuit employed^5^, and highlight the possibility for further improvements. Despite the nonidealities, the extrinsic *f*_C_ of our diodes surpass those achieved previously using different processing technologies and/or semiconductor materials, such as solution-processable metal oxides^5,7,9,19–22^, organic polymers^23–26^, organic small-molecules^27–30^, and various low-dimensional semiconductors^4,31^. Figure 5 summarizes the most important developments over the years in the area of emerging Schottky diodes technologies with the details of each study listed in Supplementary Table 4. Evidently, our self-formed IGZO Schottky diodes offer superior performance, while maintaining manufacturing simplicity and scalability. We thus believe that the self-forming nanogap method combined with FLA processing, meets all prerequisites for an alternative, rapid and mass manufacturing paradigm for large-area RF electronics that can significantly impact the emerging 5 G/6 G markets by helping to wirelessly connect as well as power the IoT device ecosystem of the future.

## Methods

### Fabrication of co-planar nanogap electrodes

First, Borofloat glass wafers (from Semiconductor wafer Inc.) underwent a sequential cleaning with DI water/acetone/isopropanol (IPA) under sonication for 10 min in each solvent. Next, Aluminum (Al) electrodes (M1) of 100 nm thickness were thermally evaporated in high vacuum (10^−6^ millibar) at a rate of 2 Å/s and patterned to the desired shape with conventional photolithography and wet etching. The M1 patterns can be done two different ways using brightfield (at the end, Al will be outer electrode) and darkfiled (Al will be inner electrode) patterning approach. For standard process, we have followed brightfield patterning however, the later method can also be used. A SAM solution that contains a 1 mM (7.8 mg) of octadecyl phosphonic acid (ODPA, purchased from Sigma–Aldrich) in 30 mL of IPA, as a solvent was prepared. The patterned substrates were immersed in the SAM solution overnight (20 h) to form a self-assembled monolayer specifically on the Al (M1) surface, but leaving the glass substrate surface without a SAM. The substrates were then rinsed with IPA, dried by nitrogen gas, and annealed at 80 °C for 10 min to remove any physisorbed ODPA molecules and excess solvents. Finally, the platinum (Pt) electrode (95 nm) with a 5 nm titanium (Ti) under-layer to promote adhesion to the glass substrate was deposited via electron-beam evaporation. Due to the poor adhesion and intrinsic stress caused by Ti-Pt (M2) films on SAM/Al surfaces, a selective removal known as self-peeling or self-forming of the Ti-Pt (M2) metal occurs. A second photolithography and wet etching step was carried out to isolate each diode, by patterning the global Al electrode (M1). To remove the ODPA SAM from M1 as well as any remaining photoresist, a UV-ozone treatment was carried out for 15 min to finally reveal the empty nanogap electrodes with a gap size typically <10 nm.

### IGZO precursor preparation, film deposition, and conversion

Firstly, 0.1 M concentration of Indium (III) nitrate hydrate (99.999% purity from Sigma–Aldrich), Gallium nitrate (III) hydrate (99.999% purity from Sigma–Aldrich) and Zinc nitrate hexahydrate (purchased from Fisher chemicals) were prepared by dissolving them separately in 2-methoxy ethanol solvent and the solutions were stirred overnight at 900 rpm. Second, IGZO solution was prepared by mixing the above solutions in a volume ratio of 5:1:3 (In:Ga:Zn) and again stirred over-night at 900 rpm. Finally, the resulting solution was filtered with a 0.2 μm PTFE syringe filter followed by the film deposition via spin-coating at 3000 rpm for 30 s over nanogap devices inside a nitrogen filled glovebox and subsequent drying at 130 °C for 10 min. The flash lamp annealing was carried out by using a Novacentrix Pulse Forge 1300. The voltage to the flash lamp was kept at 600 V, while the pulse duration was varied from 500 µs to 1250 µs and the firing rate was kept constant at 1.2 Hz. The pulse energy was varied from 4.5 J/cm^2^ to 6 J/cm^2^ for each condition, and the pulses were repeated for 20 times. The optimal diode performance was achieved at 600 V, 1.2 Hz firing rate, 750 µs pulse length, and 5.5 J/cm^2^ energy density.

### Electron microscopy

The top-view Scanning Electron Microscope (SEM) images of Al/Ti-Pt nanogap were obtained by a Helios G4 UX microscope equipped with a field emission electron source at the operating voltage of 5 kV. For cross sectional TEM images, firstly, a thin lamella was prepared with the focused ion beam (FIB) in a scanning electron microscope (Helios 400 s, FEI) equipped with a nano manipulator (Omniprobe, AutoProbe300). The sample’s surface was protected by the sequential layers of carbon and platinum deposited under electron and ion beams. The bulk of the sample was milled with a Ga ion beam to reach the depth of ca. 8–10 μm. An under-cut was made with the FIB, and the lamella was extracted from the bulk with the help of a nanomanipulator. The lamella was attached to a copper TEM grid and thinned down with FIB at 30 kV and sequentially reducing the current in the range of 2.8 nA to 93 pA. The lamella was polished with the FIB at low voltages (5 and 2 kV) to remove any possible contamination. Then, the cross-sectional images were acquired with the TEM (Titan 80–300, FEI, equipped with an electron monochromator and Gatan Imaging Filter, GIF Quantum 966) at 300 kV operating voltage. The electron energy loss spectroscopy (EELS) images were acquired in the scanning TEM (STEM) mode called spectrum imaging (SI).

### Electrical and high-frequency RF characterization

Current-voltage (I–V) characterizations of the diodes were carried out in a nitrogen-filled glovebox using the Keysight B2912A precision source/measure unit. The temperature-dependent I–V measurement was carried out in a cryogenic probe station (Lake Shore Cryotronics Inc.) in combination with a Keysight B1500A semiconductor device analyzer. The capacitance measurements were recorded with a Solartron SI 1260 impedance/gain phase analyzer at room temperature inside a nitrogen-filled glove box. High-frequency scattering parameter (S_11_) measurements were obtained (in air) through an Agilent PNA N5225A operating at 10 MHz – 50 GHz. Cascade Infinity GSG probes (ACP-40) with a pitch of 500 µm were used after a valid Short, Open, and Load (SOL) calibration on an impedance standard substrate (ISS) of 106–682. The rectifier measurements were conducted inside a vacuum-sealed chamber (1 × 10^−5^ torr) connected with a bias Tee (10 MHz to 18 GHz) through the GSG Picoprobes (from GGB industries). The output voltage was measured across a load resistor R_L_ = 10 MΩ connected with Keysight 34465 A Digital Multi Meter (DMM).

### Opto-thermal calculations and temperature profile simulations

Opto-thermal simulations were performed using COMSOL software to extract the actual temperature profile on electrode’s surfaces and at the edges of the electrodes (where the nanogap is present). The optical properties of Al, Ti, Pt and glass are obtained from Sopra database. The boundary conditions are set to convective cooling from the back surfaces and radiative cooling from the top. The ratio between outer and inner electrodes is considered as ${d}_{2}/{d}_{1}=2.5$ (for 300 µm diode), the nanogap length$l=10{{{{{\rm{nm}}}}}}$, flash light power $=5.5{{{{{\rm{J}}}}}}/{{{{{\rm{c}}}}}}{{{{{{\rm{m}}}}}}}^{2}$ fluence (${f}_{A}=1.74{{{{{\rm{J}}}}}}/{{{{{\rm{c}}}}}}{{{{{{\rm{m}}}}}}}^{2}$ absorbed), and a pulse duration of $\tau =750{{{{{\rm{\mu s}}}}}}$ were considered as the best optimized conditions for the precursor conversion.

### RF current profile simulations using HFSS

Electromagnetic simulations of diodes have been conducted using ANSYS High-Frequency Simulation Software (HFSS). Four different dimensions of the diodes are simulated. First, Borofloat glass substrate with dimensions of 3 mm × 3 mm × 1.1 mm is drawn and frequency-dependent electrical properties of the glass, which have been characterized beforehand, are assigned to the substrate. Next, the outer circular electrode made of Al (M1) is created with a diameter of 1300 um, which serves as a ground plane. The difference between the four designs is the diameter of the inner circular electrode made of Ti-Pt (M2, Ø = 100, 300, 600, and 900 µm). For Al and Pt, the bulk metal properties are selected from the HFSS library. The gap between the electrodes is 10 nm. The launching of RF input signal to the diodes has been done from the top, accurately mimicking the GSG probe with a pitch of 250 µm. After the simulation, the magnitude of the currents is plotted on the electrodes, and an identical color code and range are selected for accurate comparisons.

## Supplementary information

Supplementary Information

Description of Additional Supplementary Files

Supplementary Movie 1

## Data Availability

The data that support the findings of this study are available from the corresponding authors upon reasonable request.
