# Supplementary material for: Rapid and up-scalable manufacturing of gigahertz nanogap diodes
Source: Nat Commun. 2022 Jun 7;13:3260. doi: 10.1038/s41467-022-30876-6 (PMC9174168; doi:10.1038/s41467-022-30876-6)
Supplement: Supplementary file 1 — Supplementary Information [file 41467_2022_30876_MOESM1_ESM.pdf]

Supplementary Information

Rapid and upscalable manufacturing of gigahertz nanogap diodes

Kalaivanan Loganathan[1], Hendrik Faber[1], Emre Yengel[1], Akmaral Seitkhan[1], Azamat Bakytbekov[2], Emre Yarali[1], Begimai Adilbekova[1], Afnan AlBatati[1], Yuanbao Lin[1], Zainab Felemban[1], Shuai Yang[2], Weiwei Li[2], Dimitra G. Georgiadou[3], Atif Shamim[2], Elefterios Lidorikis[4,5] and Thomas D. Anthopoulos[1*]

[1] King Abdullah University of Science and Technology (KAUST), Division of Physical Science and Engineering, KAUST Solar Center (KSC), Thuwal 23955-6900, Saudi Arabia.

[2] King Abdullah University of Science and Technology (KAUST), Division of Computer, Electrical, and Mathematical Science and Engineering, Thuwal 23955-6900, Saudi Arabia.

[3] Electronics and Computer Science, University of Southampton, Southampton SO171BJ, UK.

[4]Department of Materials Science and Engineering, University of Ioannina, Ioannina 45110, Greece.

[5]University Research Center of Ioannina (URCI), Institute of Materials Science and Computing, 45110 Ioannina, Greece.

*Correspondence to thomas.anthopoulos@kaust.edu.sa

This word file includes:

Supplementary Texts **1-7**

Supplementary Figures **1-23**

Supplementary Table **1-4**

Supplementary References **1-39**

Supplementary Texts

ST 1. Self-Assembled Monolayer (SAM) formation on Al (M1) electrodes

The key step that enables the nanogap formation between Al (M1) and Ti-Pt (M2) electrodes is the formation of a self-assembled monolayer (SAM). Herein specifically, we used octadecylphosphonic acid (ODPA) as a SAM molecule which has the specific affinity to bind with native oxides of metals such as Al_2O_3 on Al, TiO_2 on Ti, and Cr_2O_3 on Cr. As shown in **Supplementary Fig. 1 to 2** the schematics showing the specific formation of ODPA on Al (M1) surfaces, while leaving the substrate without a SAM. Here, the M1 could be extended for other metals other than Al, such as Ti, ITO or Cr. First, The phosphonic acid head groups (-POOH) physically adsorbs on the Al (M1) surfaces. Then, gradually over time and facilitated by the solvent evaporation, they establish a chemical bond with the AlO_x native oxide, forming a monolayer of ODPA atop of Al (M1)[1]. The ODPA SAM tail end ($-CH_3$) is chemically inert and creates a hydrophobic surface characteristic that causes poor adhesion of Ti-Pt (M2) on top of these SAM layers.

ST 2. Intrinsic stress-induced self-peeling of Ti-Pt (M2) thin films

The poor adhesion of Ti-Pt (M2) on the SAM layer is a prerequisite but by itself is not enough to trigger the self-peeling process. The intrinsic stress developed during the e-beam deposition also plays a significant role in self-peeling of Ti-Pt films. In our experiments, the internal stress causes the rolling of Ti-Pt films into micro-tubes of diameter 14 µm to 24 µm and various other shapes. The delamination of the M2 (Ti-Pt) bilayer originates only in the overlapping regions between M2 and M1/SAM and is not observed elsewhere on the substrate (**Supplementary Fig. 3 to 5**). This clearly outlines the role of the ODPA SAM atop of Al that drastically reduces the adhesion and facilitates the self-peeling of stressed Ti-Pt films.

The M2 (Ti-Pt) bilayer starts delaminating immediately following the deposition process, and the flakes often loosely adhere to the surface. These flakes can be removed easily by blowing air or by immersing the substrate in acetone or other liquids with gentle agitation (**Supplementary Movie 1**). Since the stress is relieved immediately and the M2 film peels off, it is challenging to quantify the stress. There are stress measurement systems that use Stoney's modified equation and the radius of curvature of the substrate to find the stresses in thin films[2]. However, in this

measurement the films have to adhere on the substrate so that the change in radius of curvature can be measured before and after the film deposition and then can be used to elucidate the stresses. In a study by *Lee et al.* [3], the authors used this method to measure the stresses induced in Ti-Pt films (10-100 nm) deposited on flat Si/SiO_2 and found values in the range of 700-800 MPa. When the same films were instead deposited over a Si/SiO_2 mesh pattern, the bilayer metal film started to form Ti-Pt microtubes of approximately 25 µm diameter. This compares well to the microtube diameters obtained in our own experiments.

The FLX-2320-S stress measurement system uses two lasers at 670 nm and 780 nm and 4 mW to scan a substrate's surface before and after a film is deposited onto it. The lasers raster the surface and measure the change in curvature radius caused by the stress in the film. The interior chamber supports circular wafers of 8", 6", and 4" diameters. The accompanying software uses the collected radius of curvature measurements and known material constants and applies the following Stoney's equation to calculate the stress in the film.

$$\sigma = \frac{Eh^2}{(1-v)6Rt} \tag{S1}$$

Where E is Young's modulus, h is the substrate thickness, v is Poisson's ratio, R is the substrate radius of curvature, t is the film thickness, σ is the average film stress, and $\frac{E}{(1-v)}$ is the biaxial elastic modulus of the substrate. To measure the stress in our Ti-Pt thin films, we deposited them sequentially onto a Si/SiO_2 wafer and measured the stress. The deposited layers were 10 nm Ti and 90 nm Pt. The calculated stress in the Ti layer on the Si/SiO_2 substrate is 613 MPa (tensile) and the final stress when the Pt layer is deposited is 972 MPa (tensile). This agrees with stress values reported by Lee et al., and consequently our Ti-Pt films exhibit internal stresses within a similar range.

Contrary to Ti-Pt, self-peeling has not been observed for aluminum, gold, or titanium (as M2 electrodes) deposited using the same process and parameters. A possible explanation is that the residual strain induced during (electron-beam) deposition of other M2 (Al, Au and Ti) is insufficient [3, 4]. In addition to that, the Ti-Pt bilayer only self-peels at a critical thickness once the platinum layer is approximately ≥ 95 nm. At smaller thicknesses of Ti and Pt, the bilayer is too

thin, and immediate delamination is not observed. This could be due to the lack of insufficient stress at lower thickness [3, 5].

<u>ST 3.</u> <u>Geometric considerations for diodes:</u>

The co-planar nanogap electrodes designed in this work are illustrated in **Supplementary Fig. 5** where we clearly define the all the specific dimensions of the diodes in the top and cross-section schemes. The channel length (L) i.e., the size of the nanogap between the Al (M1) and Ti-Pt (M2) electrodes, also known as inter-electrode distance, is typically < 10 nm. The thickness of the electrodes, denoted as height (H), is kept as 100 nm. The inner Ti-Pt (M2) electrodes' diameter, d, ($Ø_{diode}$) is varied from 100 µm to 900 µm and the width (W) of the diodes are derived as $W = 2\pi r$, where $r = d/2$. The width (W) of the inner electrode M2 (Ti-Pt) multiplied by its thickness (H, 100 nm) defined as the active area (A) and the resulting values are ranging from 31 μm^2 to 282 μm^2. Due to the 250 µm pitch size of our GSG picoprobes (from GGB industries), smaller diodes of diameters 100 and 300 µm could be easily probed but, for larger diodes (600 µm and 900 µm diameter), a notch was added to the design to facilitate the landing of GSG probe for RF measurements.

<u>ST 4.</u> <u>Average nanogap size extraction:</u>

To elucidate the nanogap length between two metal electrodes, we have used two different approaches that are detailed below. These methods that were successfully employed in previous works allow us to calculate the average nanogap size and distribution [6, 7].

Method 1: In method 1, the software ImageJ [8] was used to load a high-resolution SEM image of a nanogap and then calibrated using the embedded scale bar to obtain the pixel-to-nanometer conversion factor. Next, using the measure function in the software, the nanogap size was measured manually, multiple times across the nanogap, as shown in **Supplementary Fig. 7a-b,** where each orange vertical bar represents one measurement. The vertical length of the each orange bar was considered as nanogap length. Using method 1, around 300 approximately equidistant vertical bars were drawn to extract the mean gap size and size distribution.

Method 2: In method 2, a digital image analysis approach proposed by Kano *et al.* [6] was used to determine the average nanogap distance between two metal electrodes. First, a high-resolution SEM image is loaded into ImageJ software and translated into a black and white image where all black pixels indicate a position that belongs to the nanogap space and all white pixels correspond

to the surrounding metal electrodes (**Supplementary Fig. 7c**). During this binary conversion, an algorithm is used to assist the user in finding the appropriate threshold value, which varies from one image to another based on the contrast and brightness of the SEM images used. Second, the outline of the nanogap space area is elucidated from the binary image using a different algorithm developed by Kano *et al.*, which allows to quantify the area S of the nanogap space as well as its perimeter outline P in pixel/nm. Both values are used to calculate the average gap size D_{ave} according to the Equation S2

$$D_{ave} = \frac{S}{0.5\,P} \tag{S2}$$

The area S of the gap is determined from the total number of pixels within the gap (black pixels). The perimeter (P) was found by removing pixels from the inside of the nanogap space, i.e., only selecting the outer pixels surrounding the nanogap space. Thus, the average gap size was calculated in units of pixels/nm and afterward converted to nanometer from the pixel-to-nm conversion factor, which is determined while calibrating the image as mentioned above in method 1. Other than the manual method 1, the procedure described by Kano *et al.* yields the average gap size but does not lead to a histogram of size distribution.

The mean nanogap size extracted by these two methods is approximately 16 nm, which is slightly higher than the 10 nm that were determined from cross-sectional TEM image as shown in **Fig 1c.** We note that both nanogap estimations, method 1 and 2, are based on SEM images and thus share the same characteristic limitations, which are due to the limits of image resolution and local contrast in SEM images. Resolving feature sizes on the order of a few nanometers is at the boundary of what the SEM technique can achieve. In addition, the local contrast at the edges of the gap may not allow an exact determination (on the pixel level) of the precise gap outline and, to a certain degree, this final placement lies in the judgment of the user. Using an automated algorithm for this purpose (as suggested by Kano *et al.*) can aid in this process. However, even in this method, the user has to adjust the level of thresholds for the accurate conversion into a binary image. Hence, some degree of uncertainty still remains to determine the precise nanogap length.

The nanogap size and distribution relies on various factors, including the properties of Al (M1) such as thickness, roughness and grain size influenced by deposition rate (since the nanogap forms along the boundary line of M1). So, these factors play a significant role in the process, and careful tuning of these parameters need to be considered.

ST 5. Opto-thermal simulation for flash lamp annealing on nanogap electrodes:

Optical properties of Al, Pt, Ti and glass materials were taken from the Sopra database [9]. For the dried IGZO precursor we assumed for simplicity that it has the same properties as the borofloat glass substrate. The thermal properties of metals and glass substrates were shown in **Supplementary Table 1**. The two contact systems were considered as follows: a) IGZO(13 nm)/Al(100 nm)/glass(1.1 mm) and b) IGZO(13 nm)/Ti (5 nm)/Pt(95 nm)/glass(1.1 mm) and calculated their absorptivity under normal incidence by 1D transfer matrix (**Supplementary Fig. 12e**). The flash lamp spectrum has shown in (**Supplementary Fig. 12f**), which is spectrally integrated corresponds to a fluence $f = 5.5\ \mathrm{J/cm^2}$ for a pulse duration of $\tau = 750$ µs (The best optimized power and pulse conditions) produces peak temperature variations in the precursor layer and the three distinct points (α,β,γ) are shown in **Fig. 2d** with the actual transient shown in **Fig. 2e**.

The temperature rise in Ti-Pt (α) is significantly higher than in Al (γ) due to the higher absorption in the former case ($\sim \times 4$) and the small heat decay length in glass $\xi = \sqrt{D\tau} \cong 21$ µm, where $D = k/\rho c_p$ is the corresponding heat diffusion coefficient. However, within the nanogap (β) the temperature is practically uniform. As shown in **Supplementary Fig. 12g**, the power deposited in each contact (when the pulse is on) comes from the product of source power (**Supplementary Fig. 12f**) and contact absorptivity (**Supplementary Fig. 12e**). The temporal profile of the absorbed heat which was used in the COMSOL thermal simulations [10] have shown in **Supplementary Fig. 12h**. The boundary conditions are convective cooling with $h_1 = 10\ \mathrm{W/m^2K}$ from the top (free convection) and $h_2 = 150\ \mathrm{W/m^2K}$ from the back surfaces (loose contact with metal holder) [11] as well as the radiative cooling from the top. Given the very short pule duty cycle ($\mathrm{DC} = \tau \times v = 0.09\%$) however, the latter has a minor role with the former determining the saturation background substrate temperature according to $\Delta T_{back} \approx v f_A/(h_1 + h_2)$. Here $f_A = \tau[p_{Al} + (2.5^2 - 1)p_{Pt}]/2.5^2 = 1.74\ \mathrm{J/cm^2}$ is the average fluence absorbed by the device for $d_2 = 2.5 \times d_1$, where p_{Al} and p_{Pt} are the heating rates of the Al and Pt/Ti contacts

respectively, as shown in **Supplementary Fig. 12h**. Overall, FLA allows targeted, fast and precise energy delivery in nanochannel (< 10 nm) within short time frame (< 10 s) leaving the substrates intact.

ST 6. Important figures of merit for RF Schottky diodes:

For RF applications, Schottky diodes are preferred over conventional PN junction diodes as they possess a low turn-on voltage, are easy to fabricate, and are compatible with a large range of semi-conductor and substrate materials [12]. There are several important figures of merit that can be used to assess Schottky diodes in RF device applications. Here, we discuss those quantities that plays a crucial role in rectifying RF input signals into DC output voltage.

ST 6a. Rectification ratio, non-linearity, and responsivity of the RF diodes:

The rectification ratio is defined as the ratio of the forward to the reverse current at the same (absolute) voltage and it is a measure of the diode's asymmetry[7, 13].

$$Rectification\ Ratio\ (V) = \left|\frac{I_F(V)}{I_R(-V)}\right| \tag{S3}$$

The non-linearity of the Schottky diode is a measure of the deviation from a linear resistor. It is defined as the ratio of the differential conductance (dI/dV) to the conductance (I/V) of the diode. A non-linearity of > 3 is preferred for RF device applications[7, 13].

$$Nonlinearity\ (V) = \frac{\frac{dI}{dV}(V)}{\frac{I\,(V)}{V}} \tag{S4}$$

Quasi-DC responsivity (or current sensitivity) is a measure of the change in the DC output current for a given RF input power. It is calculated in a small-signal approximation from the current – voltage (I-V) measurements. It is defined as the ratio of the second derivative of the I-V to the differential conductance[7, 14].

$$Responsivity\ (V) = \frac{1}{2}\,\frac{\frac{d^2I}{dV^2}(V)}{\frac{dI}{dV}(V)} \tag{S5}$$

<u>ST 6b.</u> <u>Series resistance calculation from I-V measurements:</u>

The diode's series resistance, R_S, was calculated from the *I-V* measurements using the method proposed by *Cheung et al.*[15]. The thermionic emission region of the I-V curve chosen for applying the *Cheung* method was adopted from the well-known thermionic equation as follows,

$$I = I_o\ e^{(qV|nKT)} \tag{S6}$$

where I_o is the reverse bias saturation current given as $I_o = SA^*\ T^2 e^{(q\Phi_B|KT)}$. Here, n is the ideality factor, S is the diode area (cm^2), Φ_B is the barrier height, and A^* is the effective Richardson constant (41 A/cm^2 K^2 for IGZO)[16]. From equation S5, the equation can be expanded to include the effect of series resistance R_S as

$$\frac{d(V)}{d(lnI)} = RI + \frac{n}{\beta} \tag{S7}$$

where, $\beta = q/kT$. By plotting d(V)/d(ln$I$) and fitting the linear region, R_S can be obtained from the slope and n from the intersect and we define equation H(I) as:

$$H(I) \equiv V - \frac{n}{\beta}\ln\left(\frac{I}{A_{eff}A^*T^2}\right) \tag{S8}$$

The plot of H(I) and a fit of the linear region are used to obtain a second approximation of R_S from the slope and Φ_B from the intersect.

$$H(I) = RI + n\varphi_B \tag{S9}$$

The Cheung plots, according to Eqs. (S6) and (S8) are shown in **Supplementary Fig. 14**.

ST 6c.Temperature-dependent charge transport analysis:

Temperature-dependent I-V characteristics are used to estimate the barrier height (Φ_B) and effective Richardson constant (A^*). **Supplementary Fig. 16** shows the I-V characteristics of IGZO diodes measured from 140 K to 300 K. Using this data and considering the thermionic emission model, the standard Richardson plot can be obtained by plotting $\ln(\frac{I_0}{ST^2})$ versus $\frac{1}{kT}$ and the corrected form of the plot may also be obtained by plotting $\ln(\frac{I_0}{ST^2})$ against $\frac{1}{nkT}$. However, these models do not take into account the temperature-dependence of n and Φ_B. Thus, Bhuiyan et al.[17] derived a model where the ideality factor n (T) and barrier height Φ_B (T) are given by

$$n(T) = a + \frac{b}{T} \quad \text{(S10)}$$

$$\Phi_B(T) = \Phi_{B0} - \alpha T \quad \text{(S11)}$$

Here Φ_{B0} is the apparent barrier height at T = 0, and a, b are constants, and T is temperature. Equation S10 allows to calculate the apparent Φ_{B0} but not the true flat band barrier height Φ_{Bfb},. This can be obtained by using

$$\Phi_{Bfb} = n\Phi_B - (n-1)\frac{kT}{q}\ln\frac{N_C}{N_D} \quad \text{(S12)}$$

Where N_C is the density of states in the conduction band, and the N_D is the carrier concentration. The modified saturation current then becomes

$$n(T)\ln\left(\frac{I_0}{ST^2}\right) = -\frac{1}{kT}(\Phi_B - kb\ln(A)) + a\ln(A) + \frac{\alpha}{k} \quad \text{(S13)}$$

The values for S, α and b are extracted from the plots of n (T), and Φ_B (T) versus T. Further, $n(T)\ \ln\left(\frac{I_0}{ST^2}\right)$ vs $\frac{1}{kT}$ was plotted and the best fit for the data yields a value for A = 41.6 ± 6 A cm^{-2}K^{-2} and the barrier height at T = 300 K (Φ_B) of 0.75 ± 0.2 eV has been found. The Φ_B value is in good agreement with previously reported barrier height[18].

ST 6d. Schottky barrier height and dopant concentrations

C-V measurements are considered as an alternative method to extract the Schottky barrier height. This approach is regarded as most practical as the value of flat band barrier height is determined, and the effects of image force lowering are negligible[19]. In the *C-V* data, we apply the Mott-Schottky plot, $\frac{1}{C^2} = f(V)$, to calculate the built-in voltage, V_{bi}, namely the voltage at which there is no band bending, or charge depletion that separates depletion from accumulation region, and the dopant concentration $N_{A/D}$. Here the extrinsic capacitance due to 3D coupling of the electrodes with an empty nanogap, i.e. no semiconductor material present (0.17 pF), has been subtracted from the raw data to assure the measured capacitance values come only from the complete device[20].

$$\frac{1}{C^2} = \frac{2\left(V_{bi} - V - \frac{kT}{q}\right)}{A^2 q \varepsilon \varepsilon_0 N_{A/D}} \tag{S14}$$

Where, ε is the dielectric constant of IGZO, and ε_0 is the dielectric permittivity in vacuum (ε_0 = 8.856*10^{-12} F m^{-1}).

The Schottky barrier height, Φ_B, can then be calculated from the V_{bi} and $N_{A/D}$:

$$\Phi_B = V_{bi} + \frac{kT}{q}\left(ln\frac{N_{CB}}{N_{A/D}} + 1\right) \tag{S15}$$

Where N_{CB} is the effective density of states in the conduction band and is calculated as follows:

$$N_{CB} = 2\left(\frac{2\pi m^* kT}{h^2}\right)^{3/2} \tag{S16}$$

For IGZO, given m* = $0.27m_0$ [16], and the calculated $N_{CB} = 3.5\times10^{18}$ cm^{-3}.

<u>ST 7.</u> <u>Cut-off frequency estimation:</u>

Cut off frequency of a Schottky diode is a somewhat generic term and can be referring to an intrinsic or extrinsic cut off frequency, depending on the context[12]. When the diodes are incorporated in rectifier circuits, the extrinsic cut off frequency ($f_{C,ext}$) can be determined from the output voltage progression with frequency. The output voltage is subjected to losses due to reflection, impedance mismatch, skin depth effects, and dielectric losses[14]. The intrinsic cut off frequency ($f_{C,int}$) on the other hand, measured via one port S_{11} reflection measurements, excludes those losses associated with the device and represents the theoretical upper limit. As a result, the intrinsic cut off frequency values are always higher than the extrinsic ones. There is consequently a need to elucidate both frequencies; hence we measured one port S_{11} measurement (to evaluate the intrinsic cut off frequency) and also incorporated our diodes into rectifier circuits to extract the extrinsic cut-off frequency.

The intrinsic cut-off frequency can be theoretically extracted via examining the equivalent circuit model as shown below

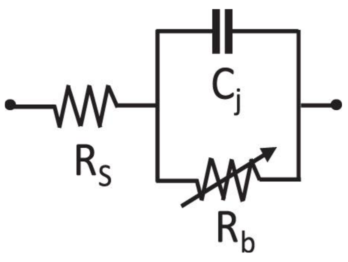

Supplementary Scheme 1 | Equivalent circuit model for a Schottky diode, showing series resistance (R_S), parallel junction capacitance (C_j) and barrier resistance (R_b).

The equation gives the output voltage of this RC circuit as

$$V_{out} = V_{in} \frac{X_C}{\sqrt{R_S^2 + X_C^2}} \qquad (S17)$$

Where the series resistance, R_S in series with nonlinear barrier resistance R_b, and the Xc is reactance associated with capacitance (C_j) given as

$$X_C = \frac{1}{\omega C_j} = \frac{1}{2\pi f C_j} \quad \text{(S18)}$$

Where f is the frequency in the equation S18**.** It is evident that at lower frequencies $X_C >> R_S$, the resistive elements mainly dominate the current transport and the rectification occurs. On the contrary, at higher frequencies, Xc << Rs, the current flow is shorted through the capacitive element, and the rectification ceases at reasonably high frequencies. The threshold frequency at which the latter happens is defined as the intrinsic cut-off frequency where the $X_C = R_S$ [12] . From f_C, the RC constant may also be determined.

$$f_{C-Schottky} = \frac{1}{2\pi R_S C_j} \quad \text{(S19)}$$

The underlying solution to find the intrinsic cut-off frequency of the Schottky diodes relies on the two essential factors, as mentioned above, series resistance (R_S) and junction capacitance (C_j). The series resistance, R_S, is due to the combination of intrinsic semiconductor resistance (R_{SP}) and the contact resistance between metal and semiconductor R_C (both Ohmic, R_{ohmic}, and Schottky contact R_{SC}). The series resistance can be calculated from either static I-V measurements using a method proposed by Cheung et al., [15] or from one port S_{11} dynamic reflection measurements. However, the frequency-dependent series resistance and capacitance values are more reliable and are widely used to extract the impedance of the diode as well as to predict the intrinsic cut-off frequency [18, 21, 22].

The extracted impedance of the Schottky diodes from S_{11} measurements, which consists of a real (series resistance, R_s) and imaginary part (reactance X_c, primarily due to capacitance C_j), is plotted against frequency. The crossover point at which the real and imaginary values meet is considered as the intrinsic cut-off frequency [18, 22], as shown in **Supplementary Fig. 22**. At this

point, the device impedance is matched with the input RF signal, and ideally, the device allows more than 90 % of the input signal. However, this does not ensure that all the signal will be transmitted to the device. This is where the losses, as mentioned above, play a significant role in rectifying the input signal.

To include such losses and re-evaluate the actual (extrinsic) cut off frequency ($f_{C,ext}$), we conducted a rectifier circuit measurement where one can directly extract the -3dB point. The -3dB point is defined as the frequency at which the output voltage, V_{OUT}, reaches $\frac{1}{\sqrt{2}} \approx 0.707$ of its peak value, V_m, where V_m is the maximum output voltage or the voltage at low frequencies. There is clear evidence that the cut off frequency drops dramatically from S_{11} to rectifier measurements due to the losses as mentioned earlier. Nevertheless, in most of the real-life applications, the device can be specifically designed to maximize the power conversion within a narrow frequency range band. From equation S19**,** it is clear that the minimization of series resistance and junction capacitance is critical for the high-frequency operation of Schottky diodes. However, from **Supplementary Table 3,** it is clearly outlined and shown that there is an option for device performance scaling in our nanogap diodes to target the specific frequency band in 5G/6G communications, radio frequency identification tags and RF wireless energy harvesting applications.

Supplementary Figures

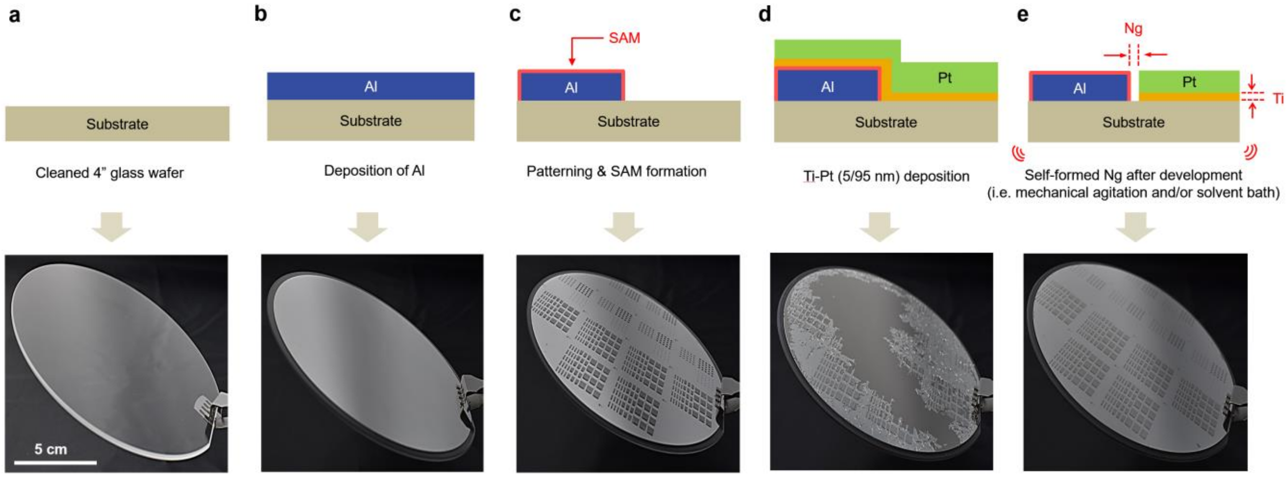

Supplementary Fig. 1 | Fabrication of self-forming nanogap electrodes. (**a-e**) Schematics, and wafer-scale photograph of each fabrication step. The patterning and selective SAM formation on Al electrodes followed by Ti-Pt deposition, resulting in the self-peeling of Ti-Pt films on Al/SAM surfaces. The final wafer (**e**) consists of arrays of circular and interdigitated electrodes (IDE) Al/Ti-Pt nanogap electrodes. The circular electrodes have inner electrode diameter ranging from 100, 300, 600 and 900 µm and the IDE electrodes have nanogap width ranging from 1cm to 5 cm.

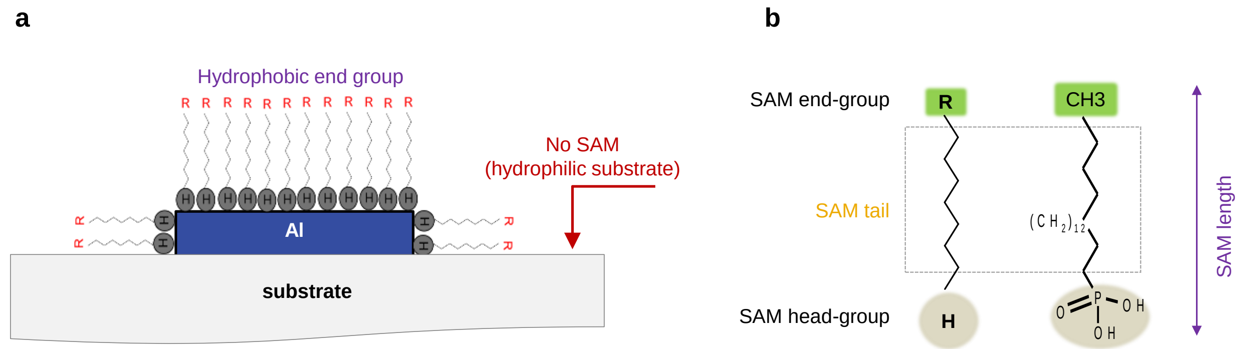

Supplementary Fig. 2 | Schematic illustration of ODPA SAM formation. (**a**) The Schematic illustration of the ODPA SAM formation on M1 (Al) electrodes via a bonding of phosphonic (-POOH) head groups of the SAM. The phosphonic head groups are specifically attached on the M1 (Al) surfaces where the thin native AlO_X layer present and the methyl ($–CH_3$) end groups are pointed outwards and organize themselves over a time. The alkyl chain and methyl end group is hydrophobic in nature and thus causes the poor adhesion to subsequently deposited layers and facilitate the self-peeling of Ti-Pt (M2) on Al/SAM surfaces. (**b**) Shows the generic molecular structure of SAM forming molecules and the specific molecule (ODPA) used in this study. The phosphonic (-POOH) head group has an affinity towards native oxides of metals such as (Al_2O_3 in Al, TiO_2 in Ti and Cr_2O_3 in Cr). Consequently, this enables the specific attachment to the surface of either Al, Ti, ITO or Cr but leaves the substrate uncovered by the SAM.

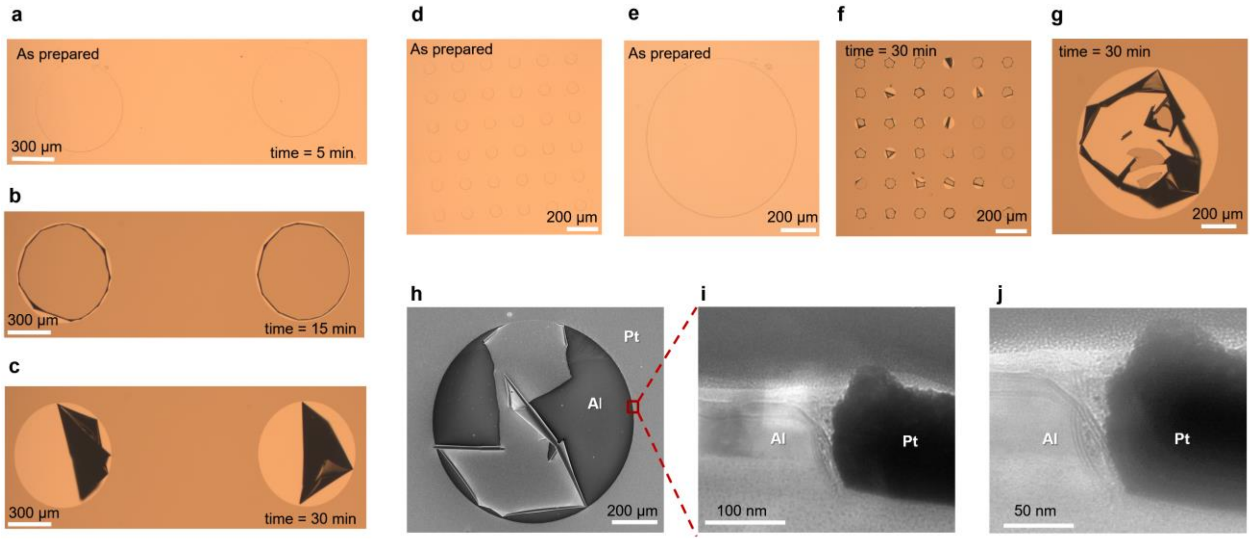

Supplementary Fig. 3 | Microscope images capturing the self-peeling process. (**a-g**) Optical microscope images showing the self-peeling of M2 (Ti-Pt) films over time in air. (**h**) SEM image displays the self-peeling of Ti-Pt films only on top of the Al/SAM surface immediately after the M2 (Ti-Pt) deposition. **(i-j)** High-resolution cross-sectional TEM images taken directly after the M2 (Ti-Pt) deposition (before immersing into acetone or any other solvents) revealing the self-peeling mechanism. Here, the metal electrodes are separated by only a few nanometers and the specific device has shown perfect isolation (current levels below 0.1 nA) when measured before cross-sectional TEM imaging and thus the nanogap length down to few nm is plausible.

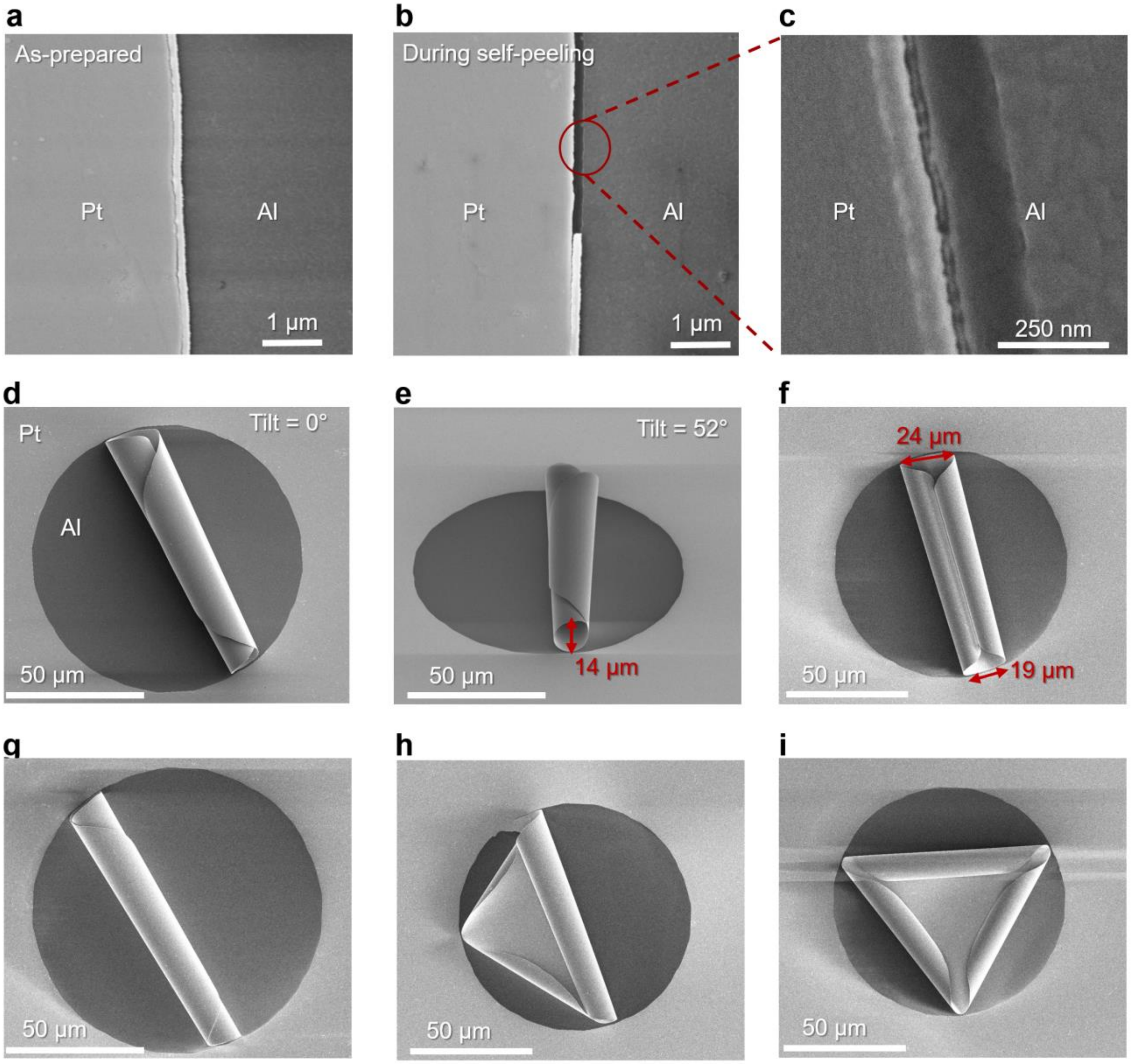

Supplementary Fig. 4 | Electron microscope images during the self-forming process: (**a**) As-deposited Ti-Pt film that has already peeled off from the Al/SAM surface, but where Ti-Pt films still remains in the gap. (**b**) Ti-Pt film that additionally has begun to partially peel off of the nanogap region. (**c**) Top-view of the self-peeled nanogap region. (**d-e**) Images of Ti-Pt films that self-rolled into micro-tubes of diameters below 30 µm, viewed at 0º and 52º tilt angle (**f-i**) Images of other possible shapes during the self-peeling process (the viewing angle is 0º).

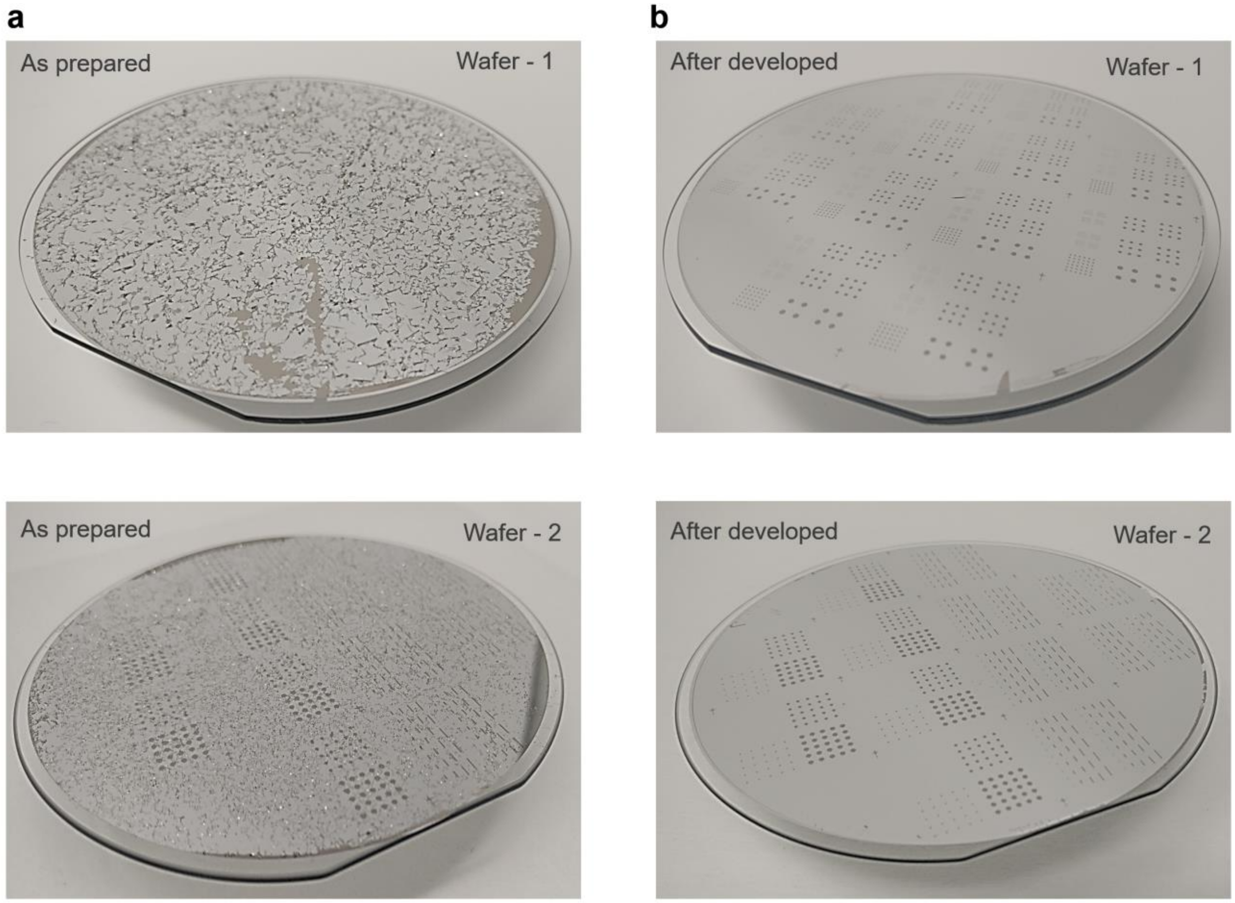

Supplementary Fig. 5 | Large area scalability of self-forming nanogap fabrication method: (**a**) Photograph of the self-peeling of Ti-Pt films which is independent of shapes and sizes of Al electrodes. Nanogap electrodes of different shapes and dimensions fabricated on a 4-inch wafer reveal that this method can be scalable and reliable for large-area applications. (**b**) Images of the wafers after immersion into acetone and subsequent cleaning/drying with a nitrogen stream. The photographs are revealing the successful fabrication of arrays of co-planar circular Al/Ti-Pt nanogap electrodes with diameters of 100, 300, 600, and 900 µm (wafer 1 and 2) and elongated bar structures of nanogap width ranging from 1 mm to 5 mm (wafer 2). This clearly show the scalability of our self-forming nanogap fabrication method in any desired shapes.

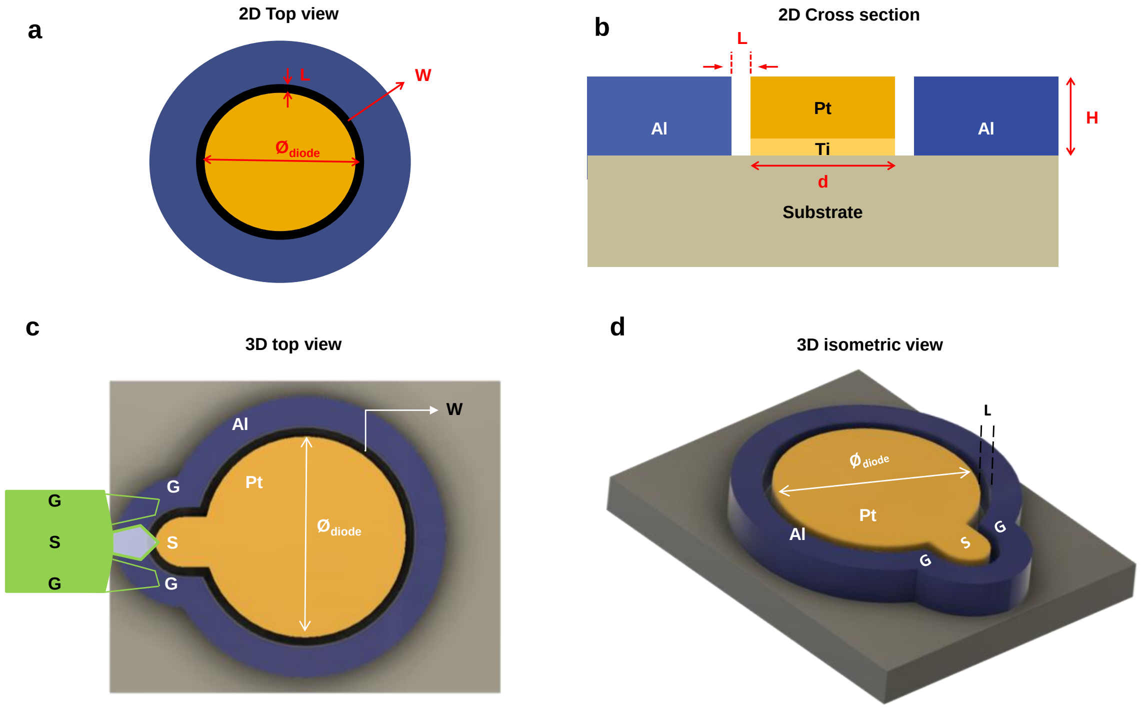

Supplementary Fig. 6 | The schematic (2D and 3D) view of co-planar Al/IGZO/Ti-Pt nanogap diodes: (**a-b**) Schematics of 2D top view and 2D cross-section view of a nanogap device which clearly outlining the various dimensions of the diodes: the nanogap length, L, electrode thickness, H, Ti-Pt (M2) electrodes diameter ($Ø_{diode}$), d, and the perimeter ($2\pi r$) of the M2 electrode defined as width, W. (**c-d**) 3D top view and 3D isometric view of the device layout clearly showing the GSG probing pads and planar nanogap between Al & Ti-Pt electrodes. We used four different diameters (100, 300, 600 and 900 µm) to investigate the diode performance with respect to the change in the active area of the diodes (ranging from 31 μm^2 to 282 μm^2) in DC and RF measurements. For RF rectifier measurements, we adopted additional notches in 600 µm and 900 µm diodes to accommodate RF probe, since the GSG probe was limited to 250 µm in pitch size (distance between ground, G, and signal, S).

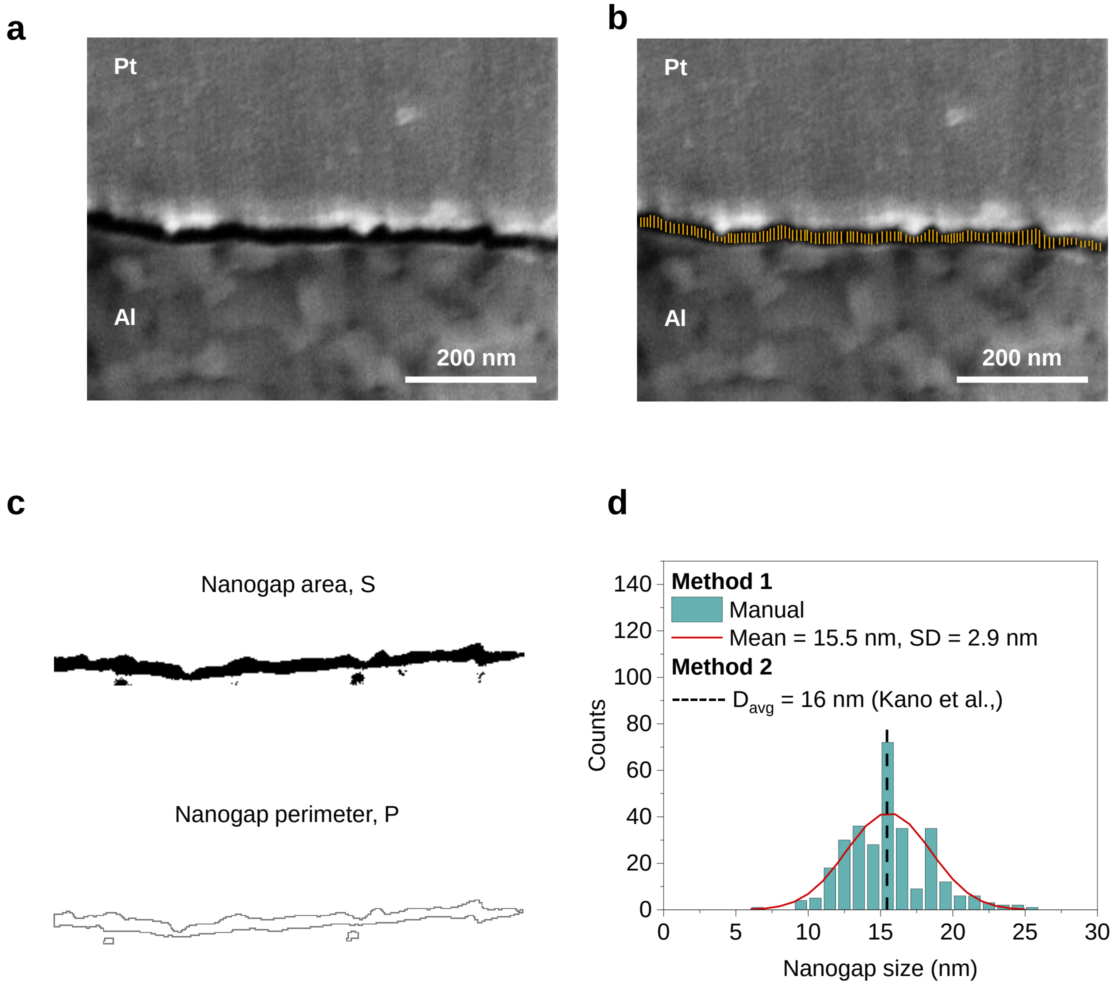

Supplementary Fig. 7 | Average nanogap size calculation. (**a**) High-resolution SEM image of Al/Ti-Pt nanogap (raw image) that is used in the following figures (**b-c**) to estimate the nanogap size. (**b**) The manual estimation of average nanogap size using ImageJ software by considering the equidistance orange lines across the nanogap area (method 1). (**c**) Showing the extraction of the nanogap area, S (upper image) and the perimeter, P (lower image) from the SEM image via a method proposed by Kano et al.,[6] and used for average nanogap size (D_{avg}) calculation (method 2). (**d**) Comparison of the mean nanogap size derived from these two different approaches as well as the gap size distribution extracted from method 1. The average nanogap size is approximately < 16 nm from both methods. SD refers to the standard deviation in the nanogap size extracted via method 1.

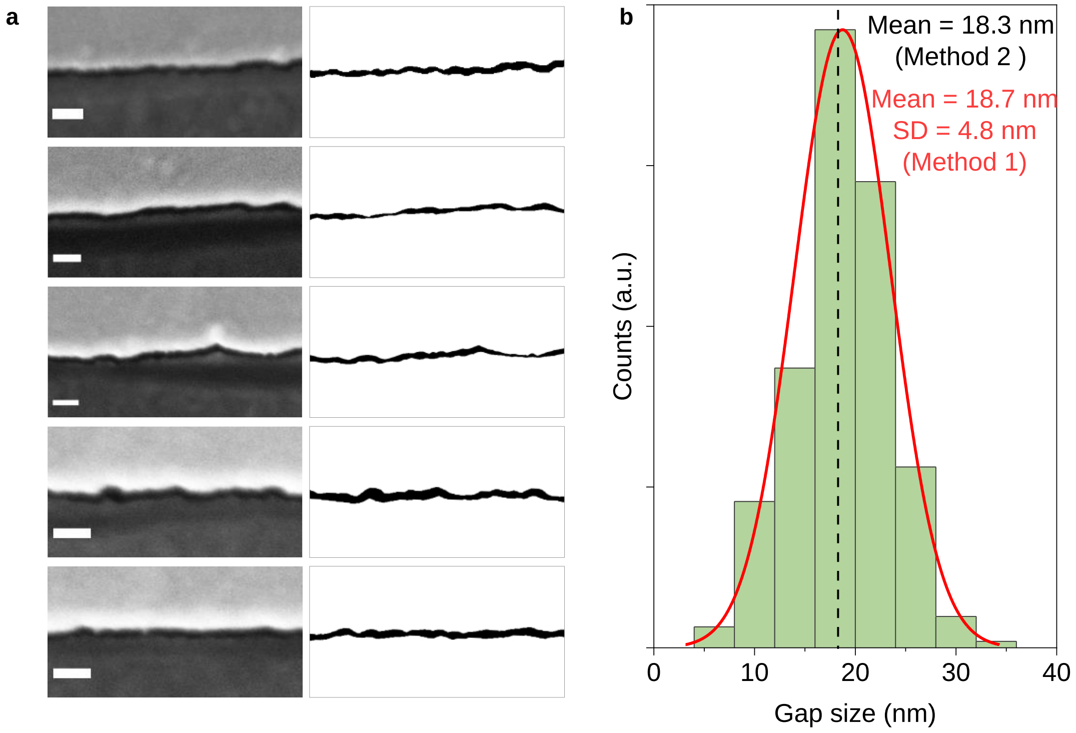

Supplementary Fig. 8 | Statistical data on Al/Ti-Pt nanogap size. **(a)** scanning electron microscope (SEM) images of five different Al/Ti-Pt nanogap devices taken from three different batches (left) and shows the nanoscale gap in dark pixels (right). The scale bar is 100 nm in each case. **(b)** The corresponding histogram that summarize the combined nanogap sizes using method 1 and 2 as described in detail in **Supplementary Text 4**. SD refers to the standard deviation in the nanogap size extracted via method 1.

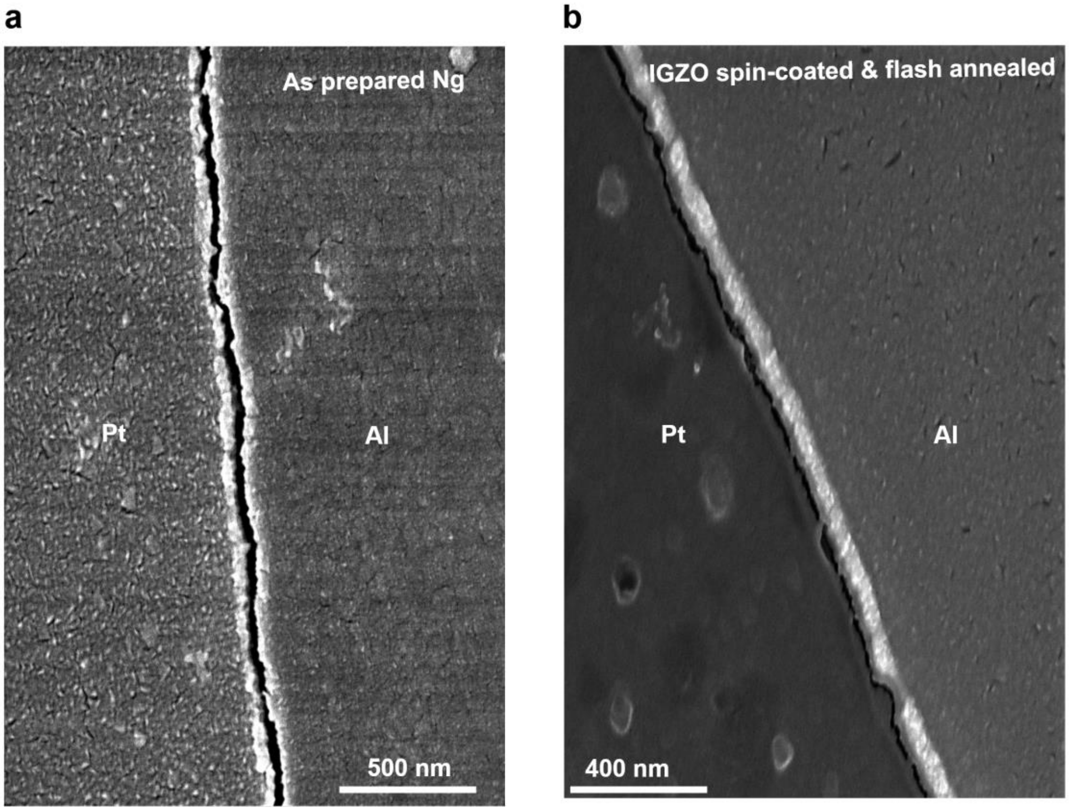

Supplementary Fig. 9 | SEM images of before and after IGZO processing. SEM images of as-prepared nanogap electrodes (**a**) and a device after IGZO spin-coating followed by flash lamp annealing reveals the filling of IGZO precursors into the nanogap channel (**b**).

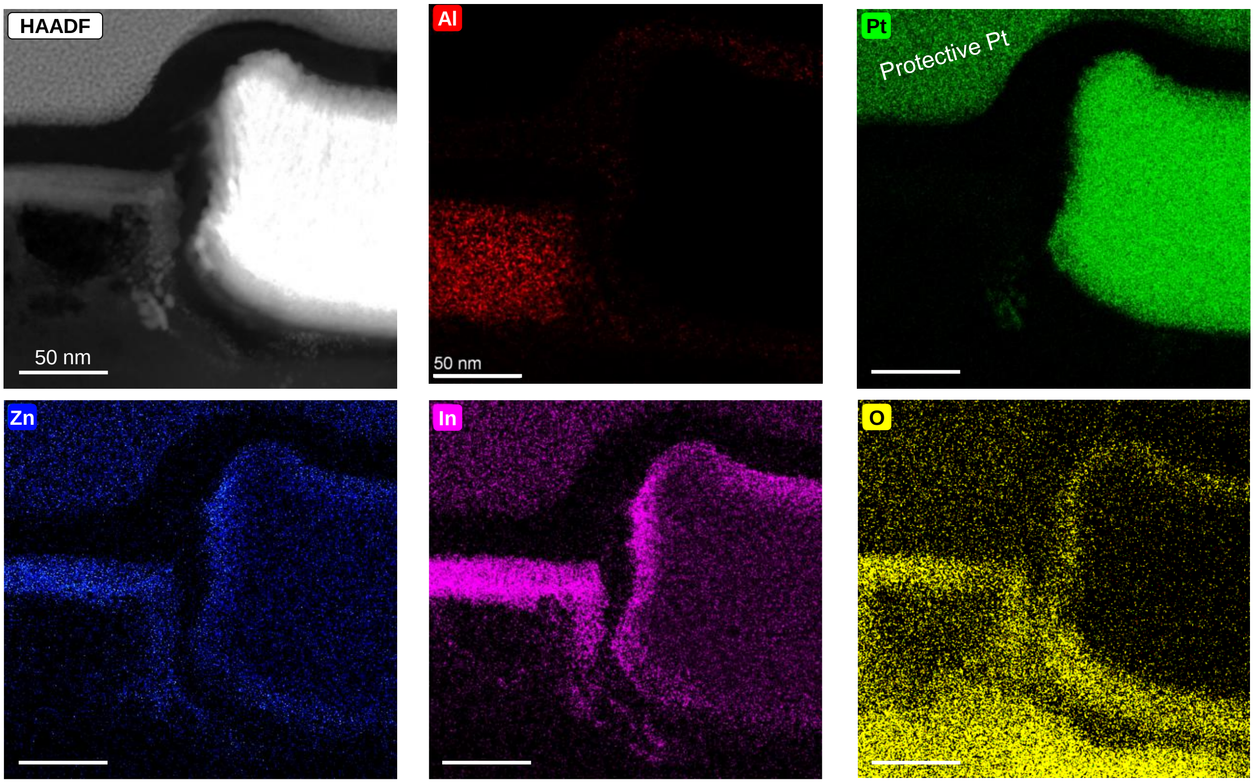

Supplementary Fig. 10 | High angle annular dark field (HAADF) image and the corresponding elemental mapping of individual Al, Pt, Zn, In and O elements.

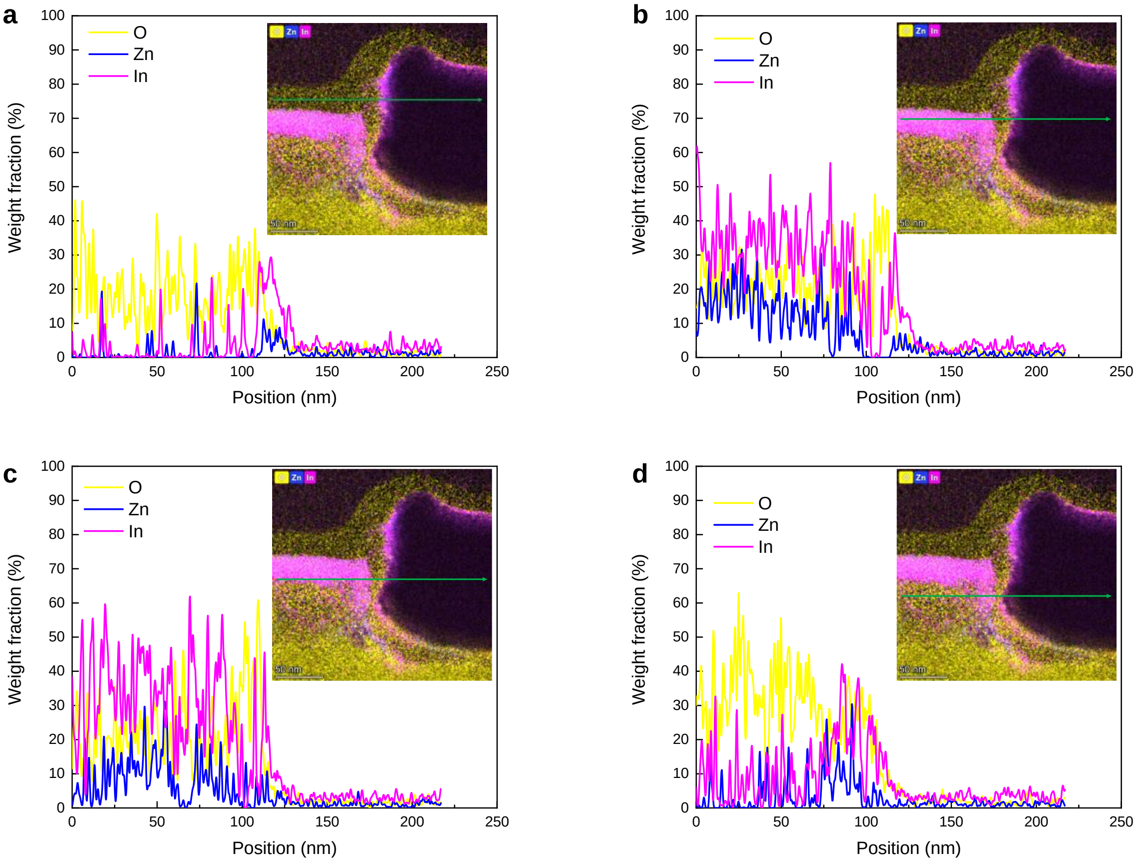

Supplementary Fig. 11 | (a-d) Zn, In and O element’s line profiles across the nanogap at varying distance from the substrate. The inset shows the combined elemental map of In, Zn, and O and the green lines indicate the regions where the line profiles were taken.

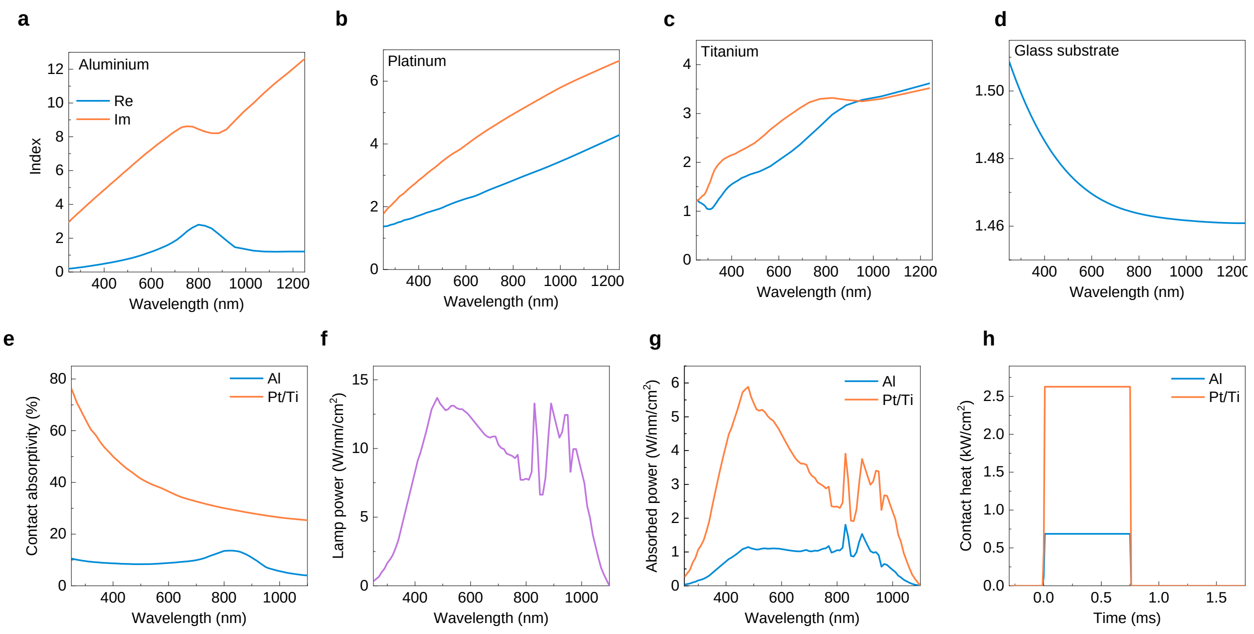

Supplementary Fig. 12 | Optical properties used in the opto-thermal simulations. (**a**) Refractive index for Aluminum, (**b**) Platinum, (**c**) Titanium and (**d**) borofloat glass obtained from Sopra database [9]. (**e**) Normal incidence absorptivity of the two metals, (**f**) flash lamp power spectrum, (**g**) power absorption rate at the two the metals and (**h**) heating profile for each metal.

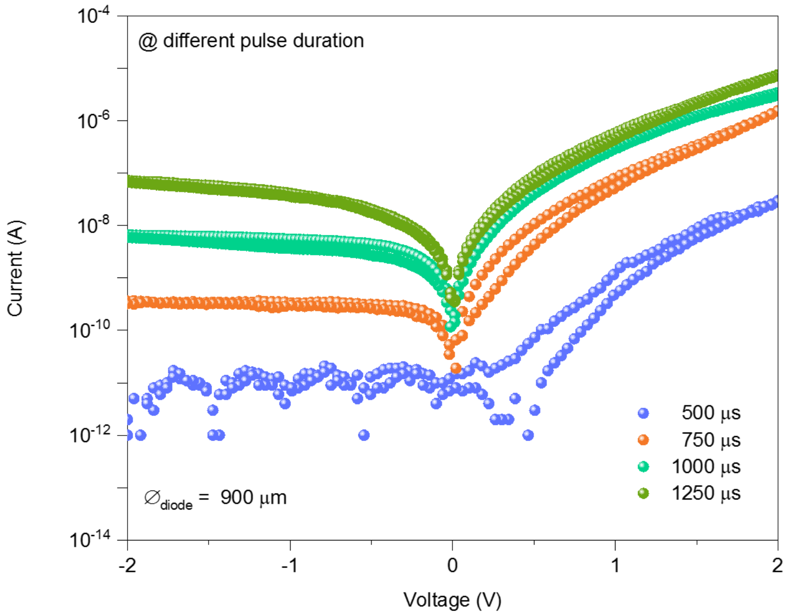

Supplementary Fig. 13 | Influence of FLA parameters on Al/IGZO/Ti-Pt diode's device performance: The I-V characteristics and optimization of FLA conditions for the best diode's performance which shows that the off current shifts to higher values at higher pulse length. The remaining parameters, such as voltage to the flash lamp, fire rate, and the number of pulses were kept at constant as 600 V, 1.2 Hz, and 20 pulses, respectively. Here, specifically 900 µm diameter Al/IGZO/Ti-Pt diodes were used to compare the impact of the FLA conditions on the diodes' performance.

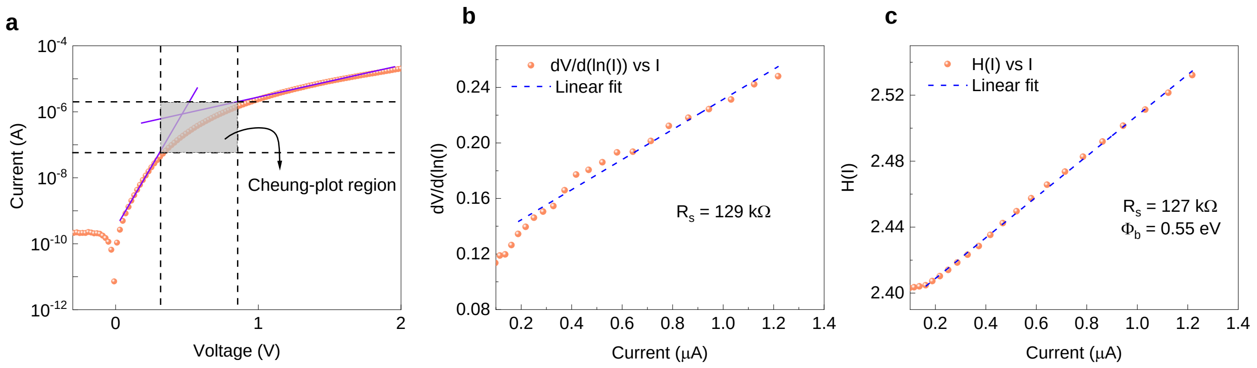

Supplementary Fig. 14 | Calculation of series resistance (R_S) and barrier height (Φ_b) from Cheung plots: (**a**) Shows the thermionic region (I-V range) used for Cheung plots. (**b**) dV/d(ln(I)) vs. diode current for the extraction of series resistance (R_S) using first-order approximation and (**c**) the H(I) vs. diode current plot for the second approximation of R_s and the barrier height (Φ_b) extraction. The results are summarized in **Supplementary Table 2**.

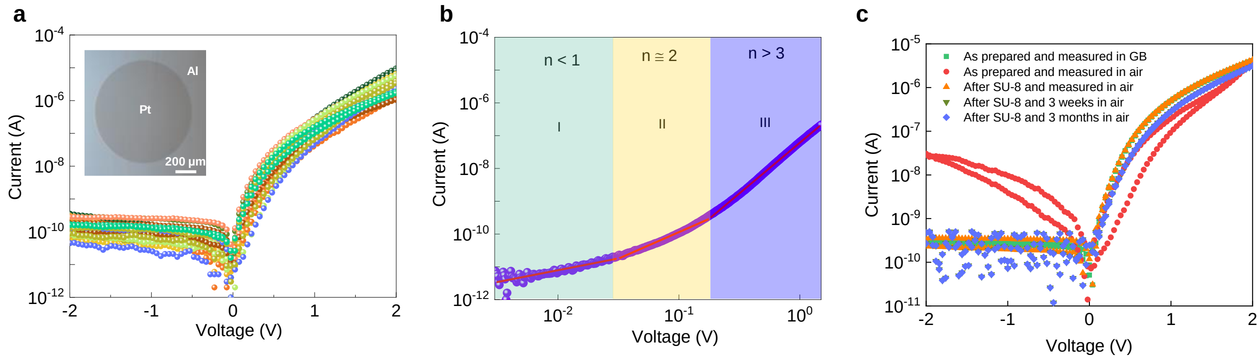

Supplementary Fig. 15 | Electrical measurements: (**a**) Consistent I-V characteristic of flash lamp annealed (FLA) Al/IGZO/Ti-Pt co-planar diodes (13 devices) from the same die, have shown near-zero turn-on voltage (100 mV) and rectifications of approximately > 10^4. The inset photograph shows a single diode of 900 µm in diameter. (**b**) logI vs. logV plot shows three distinct transport regimes (I, II and III) corresponding to tunneling, thermionic and space-charge-limited current (SCLC) as the predominant mechanism, respectively[19]. (**c**) Ambient stability of Al/IGZO/Ti-Pt diodes with and without SU-8 passivation. I-V measurements in air for SU-8 passivated diodes demonstrate that the devices are stable over three months with a negligible shift in the current-voltage characteristics. This unveils the fact that with the appropriate passivation, the planar Al/IGZO/Ti-Pt diodes can be stable in air over a prolonged time.

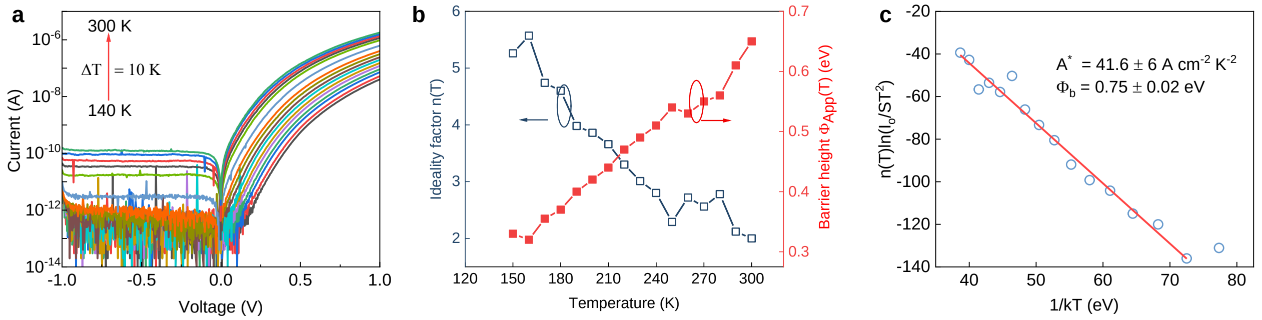

Supplementary Fig. 16 | Temperature dependent charge transport analysis: (**a**) Temperature dependent I-V characteristics of Al/IGZO/Ti-Pt diodes measured between 140 and 300 K. (**b**) The temperature dependence of the ideality factor (n) and barrier height (Φ_{App}). (**c**) The corresponding Richardson plot wherein the effective Richardson constant (A*) of 41.6 ± 6 cm^{-2} K^{-2} and the barrier height (Φ_b) of 0.75 ± 0.02 eV, were extracted. The results are summarized in **Supplementary Table 2**.

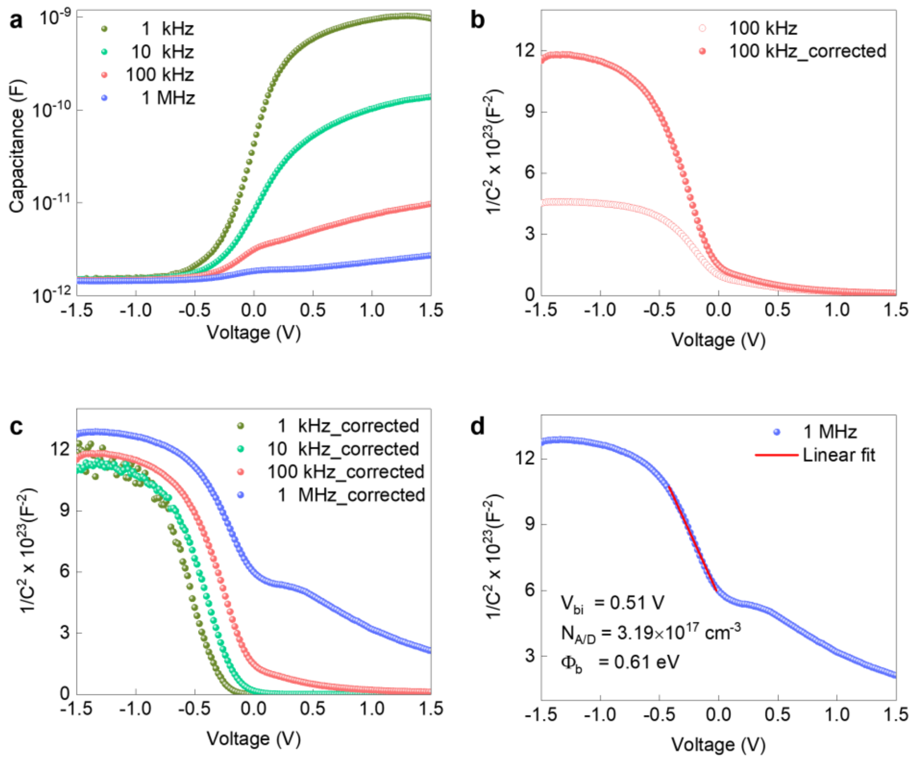

Supplementary Fig. 17 | Capacitance – Voltage (C-V) measurements: (**a**) Typical Schottky diode's C-V curve measured at a different frequency ranging from 1 kHz to 1 MHz. (**b**) The Mott-

Schottky plot for IGZO diodes, where the open circle represents the raw data and solid circles mark the data after a correction by subtraction of the corresponding extrinsic empty gap capacitance where no semiconductor material was present. (**c**) The corrected C^{-2} vs. V plot for frequencies ranging from 1 kHz to 1 MHz. (**d**) The fitting and the extraction of built-in voltage (V_{bi}) and barrier height (Φ_b). The results are summarized in **Supplementary Table 2**.

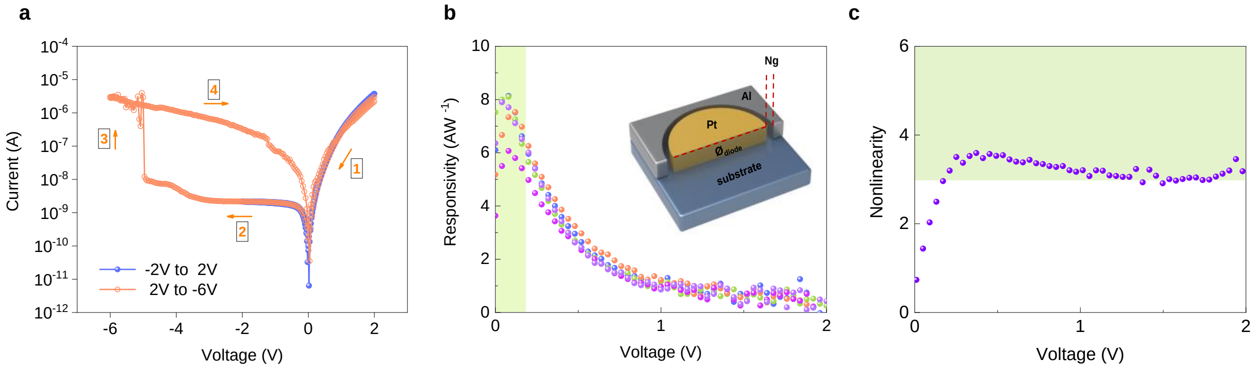

Supplementary Fig. 18 | Reverse breakdown, responsivity and non-linearity of planar Al/IGZO/Ti-Pt diodes: (a) The reverse breakdown characteristics of a representative Al/IGZO/Ti-Pt nanogap diode. The reverse breakdown observed around 5 V. (**b**) the quasi-DC current responsivity of four 900 µm diameter diodes. The near-zero voltage-current responsivity of 7-8 AW^{-1} was obtained, which is close to the thermal limit of state of art Si - RF Schottky diodes [23]. (**c**) The non-linearity curve as a function of voltage. The non-linearity is a measure of the deviation from a linear resistor and is defined as the ratio of the differential conductance (dI/dV) to the conductance (I/V) [13]. A non-linearity > 3 (marked by the green area in the graph) is preferred for high performing RF diodes.

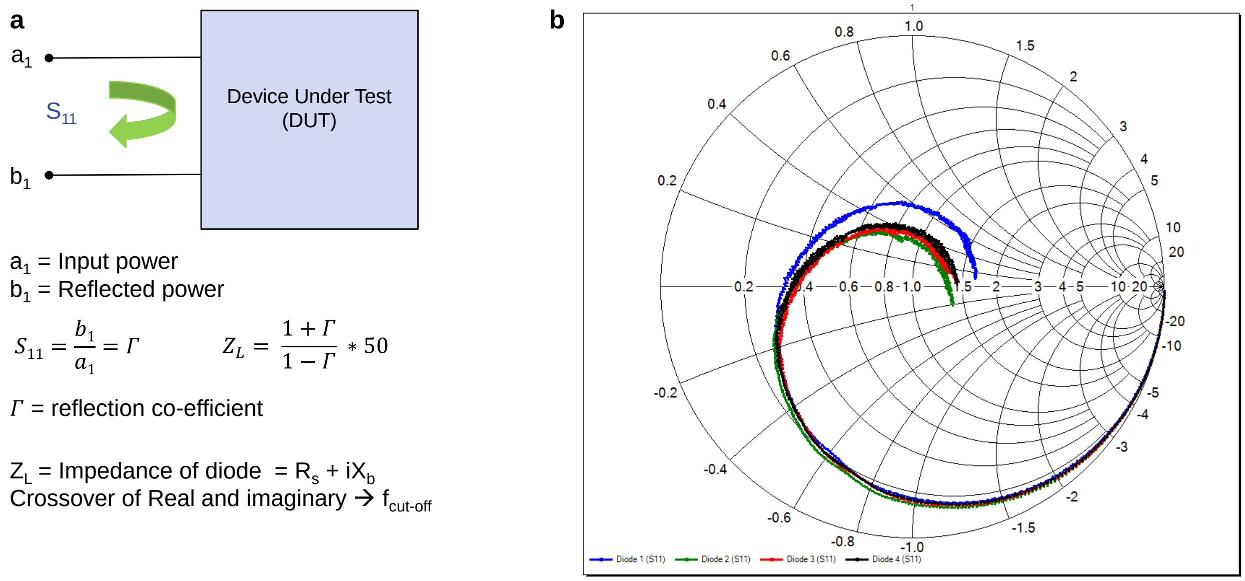

Supplementary Fig. 19 | Schematic illustration of one port S_{11} measurement and Smith chart results: (**a**) the schematic process of high-frequency one port S_{11} reflection measurements. (**b**) The Smith chart that represents the typical diode behavior of the device under test (DUT). Several Ø 900 µm devices were tested in the frequency range starting from 100 MHz to 40 GHz and have shown consistent results on Smith chart. The lower half of the circle where the plot appears reveal that the reactive component of the DUT is mainly from capacitive element (junction capacitance, C_j) of the diode.

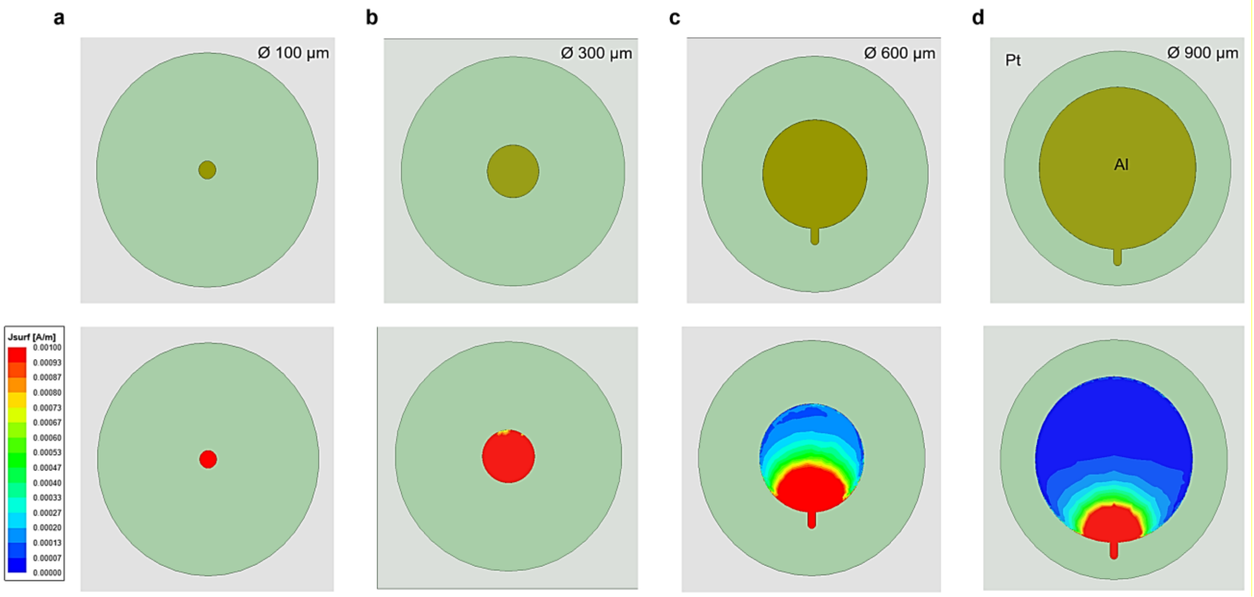

Supplementary Fig. 20 | RF current distribution profile simulation results: (**a-d**) The current distribution profile (bottom row) on Al/IGZO/Ti-Pt diodes while launching the RF signals simulated via High-Frequency Simulation Software (HFSS) on 100, 300, 600, and 900 µm diameter diodes. The current distribution for 100 and 300 µm appears to be uniform, but for 600 and 900 µm, the current is more concentrated near the probing region. This simulation reveals that at a larger device structure, launching and transmitting the RF signal becomes challenging.

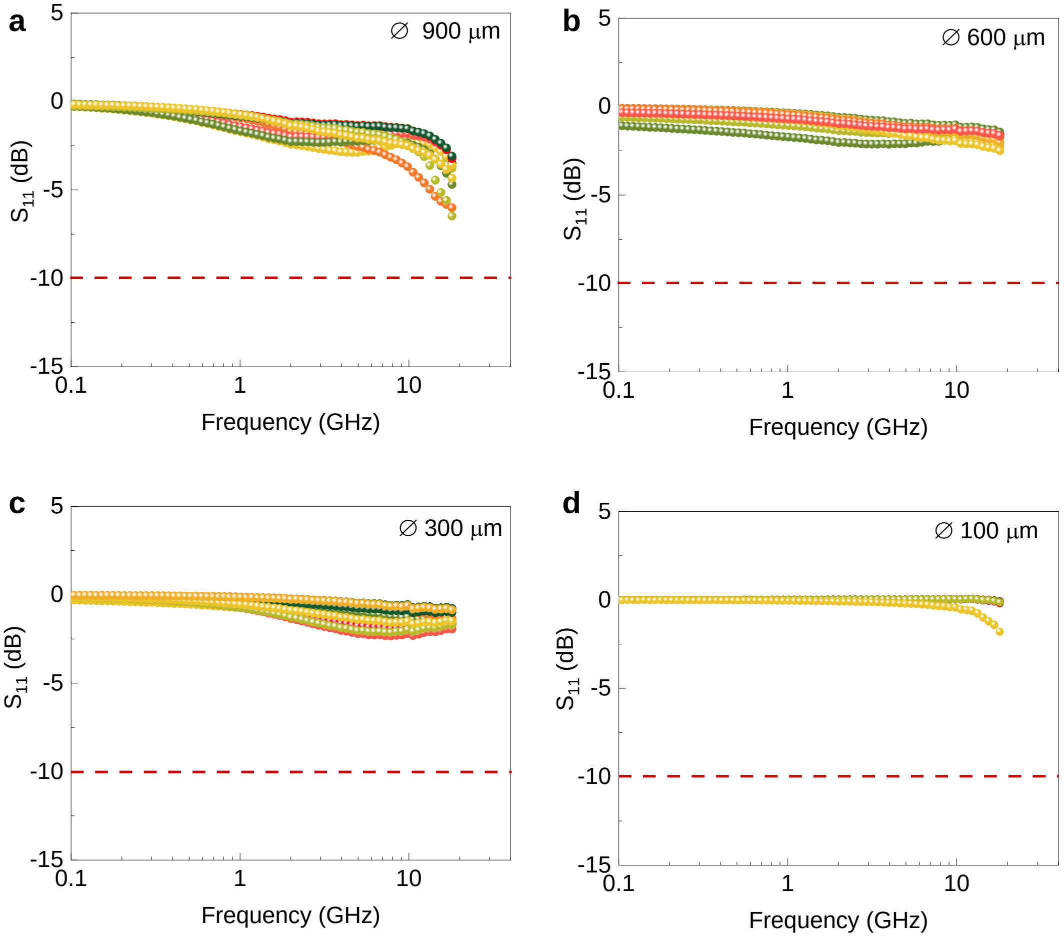

Supplementary Fig. 21 | High-frequency S_{11} measurements on several diodes: (**a-d**) the one port S_{11} measurement of 10 diodes for each diameter (900, 600, 300, and 100 μm) measured from 100 MHz to 40 GHz frequency range and showing consistent reflection results. In our co-planar devices most of the input RF signal is reflected and only small portion passes through the device. The -10 dB point (red color dotted lines) is where the 90 % of the input signal assumed to passing through the device. The corresponding impedance and intrinsic cut-off frequency estimation of the diodes are shown in the following **Supplementary Fig. 18**.

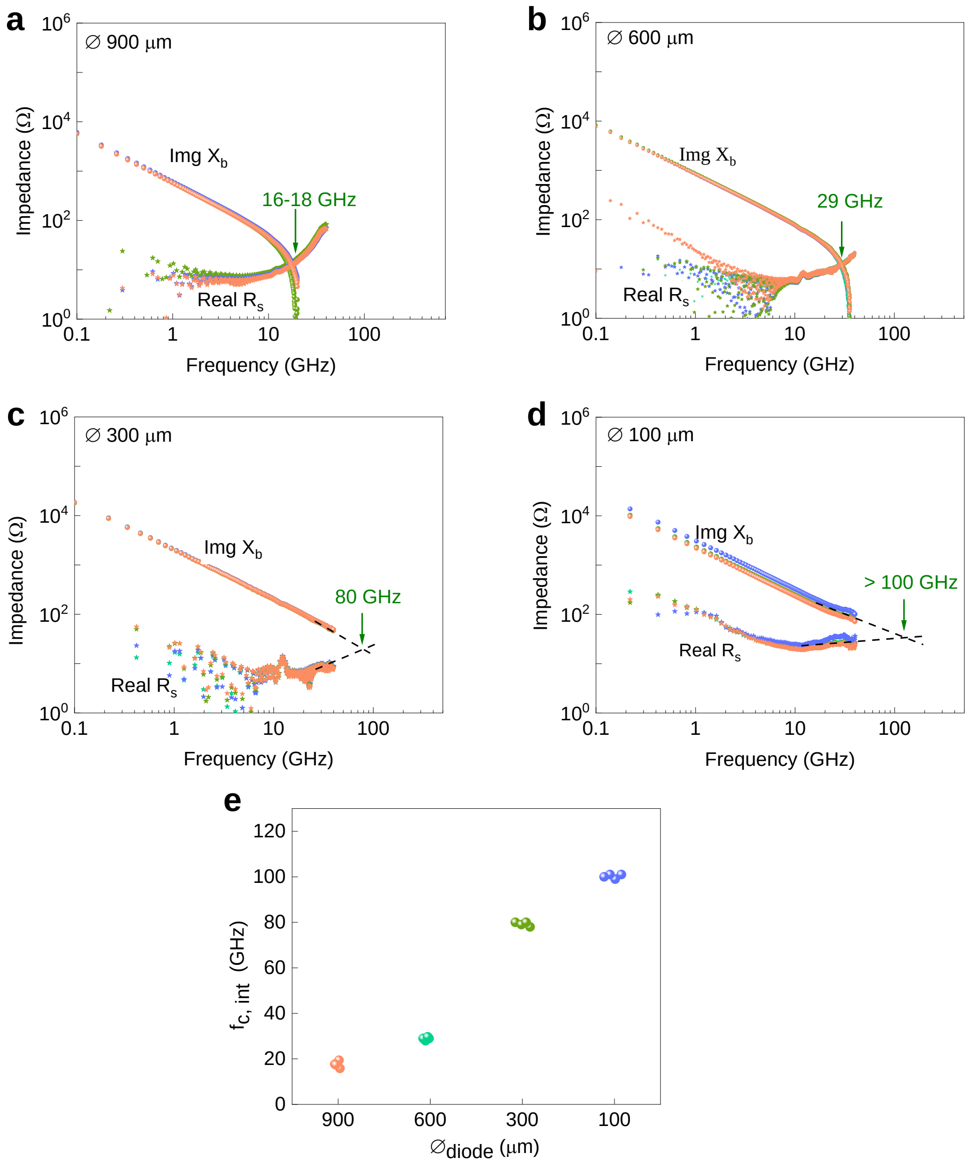

Supplementary Fig. 22 | High frequency impedance results of several diodes: (**a-d**) the impedance plot (extracted from S_{11} measurement) of four diodes for each diameter (900, 600, 300, and 100 μm). A consistent intrinsic cut-off frequency could be extracted from each diameter. (**e**) Summary of the intrinsic cut-off frequencies obtained from figure (**a-d**). The scaling trend in the intrinsic cut-off frequency, $f_{C,int}$, of the device is apparent in the plot.

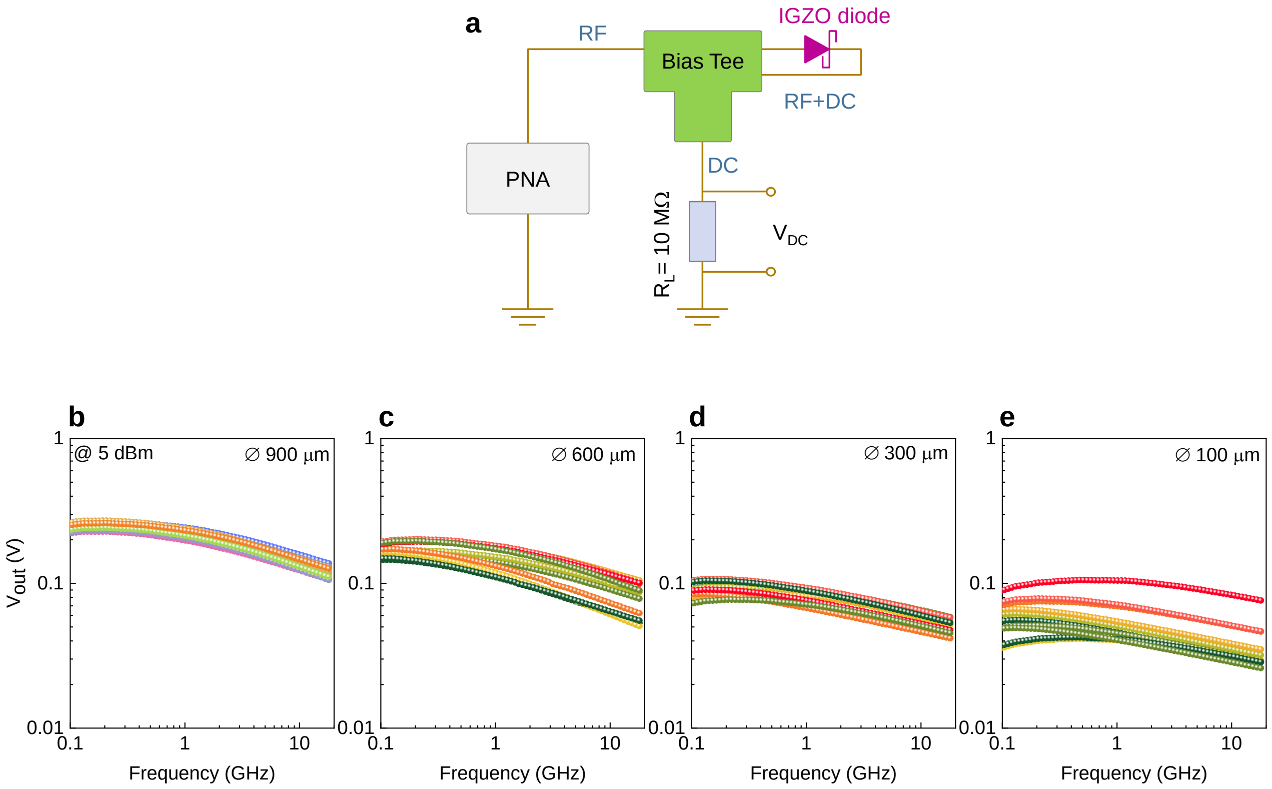

Supplementary Fig. 23 | High-Frequency rectifier measurement: (**a**) The schematic illustration of the high-frequency rectifier measurement setup, comprised of Power Network Analyzer (PNA) as a source of AC signal, the bias tee (used to allow the RF signal to nanogap diodes and divide the RF and DC signals back from the diode), and the load resistor, R_L, used to measure the rectified DC voltage from nanogap diodes. (**b-e**) The rectified DC voltage output for 900, 600, 300, and 100 µm diodes, respectively. Around ten diodes were measured for each diameter and each show a consistent voltage output. The frequency range for rectifier measurements was 100 MHz to 18 GHz. As expected, a diode's rectified output voltage increases with its diameter.

Supplementary Tables

Supplementary Table 1. Thermal properties of the materials used in the opto-thermal calculations [24].

Material	mass density ρ [g/cm^3]	specific heat capacity c_p [J/g-K]	thermal conductivity k [W/m-k]
Aluminum	2.7	0.9	210
Platinum	21.45	0.134	69
Titanium	4.5	0.528	17
Borofloat glass	2.23	0.83	1.12

Supplementary Table 2. Summary of co-planar Al/IGZO-Ti/Pt nanogap diode's junction parameters from I-V, I-V-T and C-V measurements.

**Φ_B (eV)**				**Series resistance R_S (kΩ)**		**Richardson constant**	**N_D**
**C-V**	**I-V-T**	**Cheung *et al.***	**n**	***d*V/*d*(ln(I) vs I**	**H(I) vs I**	**A*(A cm^{-2} K^{-2})**	**(cm^{-3})**
0.61	0.75	0.55	1.9	129	127	41.6 ± 6	3.1 x 10^{17}

Supplementary Table 3. Summary of co-planar Al/IGZO/Ti-Pt diode's high-frequency characteristics.

**Parameters**	**Diameter (μm)**	**f_C (GHz) (from S11 measurement)**	***R* (Ω) (from Z(Re) at f_C)**	***C* (pF) (from Z(Im) at f_C)**	**τ_{RC} (ps)**
Al/IGZO/Ti-Pt	900	17.5	13.09	0.68	9.3
	600	29	12.69	0.25	3.1
	300	79 (extrapolated)	10.4	0.20	2.1
	100	>100 (extrapolated)	20.3	0.05	1.01

Supplementary Table 4. Summary of state-of-the-art high-frequency Schottky diodes on different materials, processing routes, and device structure reported in the literature. These data are summarized in the bar chart **Fig. S21**.

Material / Diode structure	Deposition method / process	Intrinsic/extrinsic cut-off frequency	Year	Ref
Metal Oxides				
**Ag/ZnO/Al** (Vertical device on PET substrate)	Gravure printing (solution process)	*NA / 13.56 MHz	2012	25
**Al/ZnO/Au** (Co-planar device on PEN substrate)	Spin coating (solution process)	NA / 20 MHz	2016	26
**ITO/IGZO/Cu2O/Pt** (Vertical device on PET substrate)	Sputtering (PVD process)	NA / 27 MHz	2014	27
**Al/IGZO/Pt** (Vertical device on glass and PET)	Sputtering (PVD process)	16.7 GHz on glass, 6.3 GHz on PET / 2.45 GHz on PET	2015	18
**Pd/IGZO/Mo** (Vertical device on glass substrate)	Sputtering (PVD process)	1.8 GHz / 1.1 GHz<br>3 GHz / 1.5 GHz	2013<br>2014	28<br>29
**Ti-Pt/IGZO/Ti-Pt** (Vertical device on acrylate subst.)	Sputtering (PVD process)	1 GHz / NA	2020	30
**Al/IGZO/Au** (Co-planar on glass substrate)	Sputtering (PVD process)	NA / 6.4 GHz	2020	31
**Al/ZnO/Au** and **Al/IGZO/Ti-Pt** (Co-planar on glass substrate)	Spin coating (Solution process)	> 100 GHz / 5.7 GHz<br>> 100 GHz / 47 GHz	2020	[7]&This work
Small molecules				
**Al/Pentacene/Au** (Vertical device on glass substrate)	Thermal evaporation (PVD process)	NA / 1.24 GHz	2015	32
**Al/C60/WO3/Al (vertical device)** (Vertical device on glass substrate)	Thermal evaporation (PVD process)	NA / 700 MHz	2010	33
**Al/C60:Polystyrene/Au** (Co-planar device on glass substrate)	Spin coating (Solution process)	NA / 400 MHz	2016	26
**Au/PEDOT: PSS/Pentacene/Al** (Vertical device on Si substrate)	Thermal evaporation (PVD process)	NA / 50 MHz	2005	34
**Al/Pentacene (double layer)/Au** (Vertical device on PEN substrate)	Thermal evaporation (PVD process)	NA / 13.56 MHz	2008	35
Polymers				
**Cu/P3HT/Ag** (Vertical device on glass substrate)	Gravure printing (Solution process)	NA / 10 MHz		36
**IZO/PEDOT:PSS/P3HT/Al** (Vertical device on glass substrate)<br>**IZO/PEDOT:PSS/P3HT/PQT12/Al** (Vertical device on glass)	Spin coating (Solution process)	NA / 5 MHz<br>NA / 13.56 MHz	2011	37
**Cu/PTAA/Ag** (Vertical device on PET)	Gravure printing (Solution process)	NA / 13.56 MHz	2013	38
**ITO/PEDOT:PSS/TFB/Al** (Vertical device on glass)	Spin coating (Solution process)	NA / 13.56 MHz	2014	39
Nanomaterials (2 D and 1 D)				
**Pt/p-WSe2/ITO** (Vertical device on glass substrate)	Mechanical exfoliation and ALD process	27 GHz / > 10 GHz	2020	22
**Pd/MoS2/Au** (Planar device on Kapton substrate)	Mechanical exfoliation and E-beam lithography	>10 GHz / 10 GHz	2019	21

Supplementary References

1. Beesley DJ, Semple J, Krishnan Jagadamma L, Amassian A, McLachlan MA, Anthopoulos TD, *et al.* Sub-15-nm patterning of asymmetric metal electrodes and devices by adhesion lithography. *Nat Commun* 2014, **5:** 3933.

2. Pureza JM, Lacerda MM, De Oliveira AL, Fragalli JF, Zanon RAS. Enhancing accuracy to Stoney equation. *Appl Surf Sci* 2009, **255**(12)**:** 6426-6428.

3. Lee K-N, Seo Y-T, Lee M-H, Jung S-W, Kim Y-K, Kim J-M, *et al.* Stress-induced self-rolled metal/insulator bifilm microtube with micromesh walls. *J Micromech Microeng* 2013, **23**(1)**:** 015003.

4. Mei Y, Huang G, Solovev AA, Ureña EB, Mönch I, Ding F, *et al.* Versatile Approach for Integrative and Functionalized Tubes by Strain Engineering of Nanomembranes on Polymers. *Adv Mater* 2008, **20**(21)**:** 4085-4090.

5. R.Abermann. Measurements of the intrinsic stress in thin metal films. *Vacuum* 1990, **41**(4-6)**:** 1279-1282.

6. Kano S, Kawazu T, Yamazaki A, Fujii M. Digital image analysis for measuring nanogap distance produced by adhesion lithography. *Nanotechnology* 2019, **30**(28)**:** 285303.

7. Georgiadou DG, Semple J, Sagade AA, Forstén H, Rantakari P, Lin Y-H, *et al.* 100 GHz zinc oxide Schottky diodes processed from solution on a wafer scale. *Nat Electronics* 2020, **3**(11)**:** 718-725.

8. Schindelin J, Arganda-Carreras I, Frise E, Kaynig V, Longair M, Pietzsch T, *et al.* Fiji: an open-source platform for biological-image analysis. *Nat Methods* 2012, **9**(7)**:** 676-682.

9. SOPRA database website. Available from: http://www.sspectra.com/sopra.html.

10. COMSOL website. Available from: https://www.comsol.com/

11. Nugraha MI, Yarali E, Firdaus Y, Lin Y, El-Labban A, Gedda M, *et al.* Rapid Photonic Processing of High-Electron-Mobility PbS Colloidal Quantum Dot Transistors. *ACS Appl Mater Interfaces* 2020, **12**(28)**:** 31591-31600.

12. Semple J, Georgiadou DG, Wyatt-Moon G, Gelinck G, Anthopoulos TD. Flexible diodes for radio frequency (RF) electronics: a materials perspective. *Semicond Sci Technol* 2017, **32**(12)**:** 123002.

13. Periasamy P, Guthrey HL, Abdulagatov AI, Ndione PF, Berry JJ, Ginley DS, *et al.* Metal-insulator-metal diodes: role of the insulator layer on the rectification performance. *Adv Mater* 2013, **25**(9)**:** 1301-1308.

14. Pozar MD. *Microwave Engineering*, 2012.

15. Cheung SK, Cheung NW. Extraction of Schottky diode parameters from forward current-voltage characteristics. *Appl Phy Lett* 1986, **49**(2)**:** 85-87.

16. Zhang J, Wang H, Wilson J, Ma X, Jin J, Song A. Room Temperature Processed Ultrahigh-Frequency Indium-Gallium–Zinc-Oxide Schottky Diode. *IEEE Electron Device Lett* 2016, **37**(4)**:** 389-392.

17. Bhuiyan ASM, A.; Esteve, D. A New Richardson Plot for Non-Ideal Schottky Diodes. *Thin Solid Films* 1988,, **161:** 93−100.

18. Zhang J, Li Y, Zhang B, Wang H, Xin Q, Song A. Flexible indium-gallium-zinc-oxide Schottky diode operating beyond 2.45 GHz. *Nat Commun* 2015, **6:** 7561.

19. Semple J, Rossbauer S, Anthopoulos TD. Analysis of Schottky Contact Formation in Coplanar Au/ZnO/Al Nanogap Radio Frequency Diodes Processed from Solution at Low Temperature. *ACS Appl Mater Interfaces* 2016, **8**(35)**:** 23167-23174.

20. de Vries DK, Stelmaszyk P, Wieck AD. Intrinsic and extrinsic capacitances of in-plane-gated transistors. *J Appl Phys* 1996, **79**(10)**:** 8087-8090.

21. Zhang X, Grajal J, Vazquez-Roy JL, Radhakrishna U, Wang X, Chern W, *et al.* Two-dimensional MoS2-enabled flexible rectenna for Wi-Fi-band wireless energy harvesting. *Nature* 2019, **566**(7744)**:** 368-372.

22. Yang SJ, Park KT, Im J, Hong S, Lee Y, Min BW, *et al.* Ultrafast 27 GHz cutoff frequency in vertical WSe2 Schottky diodes with extremely low contact resistance. *Nat Commun* 2020, **11**(1)**:** 1574.

23. Zhang X, Grajal J, López-Vallejo M, McVay E, Palacios T. Opportunities and Challenges of Ambient Radio-Frequency Energy Harvesting. *Joule* 2020, **4**(6)**:** 1148-1152.

24. Matweb website. Available from: http://www.matweb.com/

25. Park H, Kang H, Lee Y, Park Y, Noh J, Cho G. Fully roll-to-roll gravure printed rectenna on plastic foils for wireless power transmission at 13.56 MHz. *Nanotechnology* 2012, **23**(34)**:** 344006.

26. Semple J, Rossbauer S, Burgess CH, Zhao K, Jagadamma LK, Amassian A, *et al.* Radio Frequency Coplanar ZnO Schottky Nanodiodes Processed from Solution on Plastic Substrates. *Small* 2016, **12**(15)**:** 1993-2000.

27. Chen W-C, Hsu P-C, Chien C-W, Chang K-M, Hsu C-J, Chang C-H, *et al.* Room-temperature-processed flexible n-InGaZnO/p-Cu2O heterojunction diodes and high-frequency diode rectifiers. *J Phys D Appl Phys* 2014, **47**(36)**:** 365101.

28. Chasin A, Nag M, Bhoolokam A, Myny K, Steudel S, Schols S, *et al.* Gigahertz Operation of a-IGZO Schottky Diodes. *IEEE Trans Electron Devices* 2013, **60**(10)**:** 3407-3412.

29. Chasin A, Volskiy V, Libois M, Myny K, Nag M, Rockele M, *et al.* An Integrated a-IGZO UHF Energy Harvester for Passive RFID Tags. *IEEE Trans Electron Devices* 2014, **61**(9)**:** 3289-3295.

30. Guerrero E, Polednik A, Ecker M, Joshi-Imre A, Choi W, Gutierrez-Heredia G, *et al.* Indium–Gallium–Zinc Oxide Schottky Diodes Operating across the Glass Transition of Stimuli-Responsive Polymers. *Adv Electron Mater* 2020, **6**(4)**:** 1901210.

31. Wyatt-Moon G, Niang KM, Rider CB, Flewitt AJ. Air Stable Indium-Gallium-Zinc-Oxide Diodes With a 6.4 GHz Extrinsic Cutoff Frequency Fabricated Using Adhesion Lithography. *IEEE Electron Device Lett* 2020, **41**(1)**:** 175-178.

32. Kang C-m, Wade J, Yun S, Lim J, Cho H, Roh J, *et al.* 1 GHz Pentacene Diode Rectifiers Enabled by Controlled Film Deposition on SAM-Treated Au Anodes. *Adv Electron Mater* 2016, **2**(2)**:** 1500282.

33. Im D, Moon H, Shin M, Kim J, Yoo S. Towards gigahertz operation: ultrafast low turn-on organic diodes and rectifiers based on C60 and tungsten oxide. *Adv Mater* 2011, **23**(5)**:** 644-648.

34. Steudel S, Myny K, Arkhipov V, Deibel C, De Vusser S, Genoe J, *et al.* 50 MHz rectifier based on an organic diode. *Nat Mater* 2005, **4**(8)**:** 597-600.

35. Myny K, Steudel S, Vicca P, Genoe J, Heremans P. An integrated double half-wave organic Schottky diode rectifier on foil operating at 13.56 MHz. *Appl Phy Lett* 2008, **93**(9)**:** 093305.

36. Lilja KE, Bäcklund TG, Lupo D, Hassinen T, Joutsenoja T. Gravure printed organic rectifying diodes operating at high frequencies. *Org Electron* 2009, **10**(5)**:** 1011-1014.

37. Lin C-Y, Tsai C-H, Lin H-T, Chang L-C, Yeh Y-H, Pei Z, *et al.* High-frequency polymer diode rectifiers for flexible wireless power-transmission sheets. *Org Electron* 2011, **12**(11)**:** 1777-1782.

38. Heljo PS, Li M, Lilja KE, Majumdar HS, Lupo D. Printed Half-Wave and Full-Wave Rectifier Circuits Based on Organic Diodes. *IEEE Trans Electron Devices* 2013, **60**(2)**:** 870-874.

39. Altazin S, Clerc R, Gwoziecki R, Verilhac J-M, Boudinet D, Pananakakis G, *et al.* Physics of the frequency response of rectifying organic Schottky diodes. *J Appl Phys* 2014, **115**(6)**:** 064509.
