# Supplementary material for: Rapid and up-scalable manufacturing of gigahertz nanogap diodes
Source: Nat Commun. 2022 Jun 7;13:3260. doi: 10.1038/s41467-022-30876-6 (PMC9174168; doi:10.1038/s41467-022-30876-6)
Supplement: Supplementary file 2 — Description of Additional Supplementary Files [file 41467_2022_30876_MOESM2_ESM.pdf]

Description of Additional Supplementary Files

File Name: Supplementary Movie 1
Description: The movie shows each processing step involved in the manufacturing of the co-planar Al/Ti-Pt nanogap electrodes. The self-peeling (delamination) of Ti-Pt (M2) films occurred specifically on regions of the substrate containing the Al/SAM (metal 1) electrode. The complete removal of remaining M2 metal is achieved by immersing the substrate into acetone and the application of gentle mechanical agitation. Alternatively, spontaneous delamination of M2 can be instigated by sonication in a liquid bath (i.e. Iso-Propyl Alcohol, or DI water) for approximately 1 min or by blowing a stream of nitrogen or air.
